# Risk factors for self‐reported insufficient milk during the first 6 months of life: A systematic review

**DOI:** 10.1111/mcn.13353

**Published:** 2022-03-28

**Authors:** Sofia Segura‐Pérez, Linda Richter, Elizabeth C. Rhodes, Amber Hromi‐Fiedler, Mireya Vilar‐Compte, Misikir Adnew, Kate Nyhan, Rafael Pérez‐Escamilla

**Affiliations:** ^1^ Community Nutrition Unit Hispanic Health Council Hartford Connecticut USA; ^2^ DSI‐NRF Centre of Excellence in Human Development University of the Witwatersrand, Office 154 School of Public Health Johannesburg South Africa; ^3^ Department of Social and Behavioral Sciences Yale School of Public Health New Haven Connecticut USA; ^4^ Department of Public Health Montclair State University Montclair New Jersey USA; ^5^ Department of Health Policy and Management Yale School of Public Health New Haven Connecticut USA; ^6^ Harvey Cushing/John Hay Whitney Medical Library Yale University New Haven Connecticut USA

**Keywords:** breastfeeding, delayed onset of lactation, health care system, cesarean section, insufficient milk, maternal obesity, risk factors, systematic review

## Abstract

The objective of this systematic review was to identify multifactorial risk factors for self‐reported insufficient milk (SRIM) and delayed onset of lactation (DOL). The review protocol was registered a priori in PROSPERO (ID# CDR42021240413). Of the 120 studies included (98 on SRIM, 18 on DOL, and 4 both), 37 (31%) studies were conducted in North America, followed by 26 (21.6%) in Europe, 25 (21%) in East Asia, and Pacific, 15 (12.5%) in Latin America and the Caribbean, 7 (6%) in the Middle East and North Africa, 5 (4%) in South Asia, 3 (2.5%) in Sub‐Saharan Africa, and 2 (1.7%) included multiple countries. A total of 79 studies were from high‐income countries, 30 from upper‐middle‐income, 10 from low‐middle‐income countries, and one study was conducted in a high‐income and an upper‐middle‐income country. Findings indicated that DOL increased the risk of SRIM. Protective factors identified for DOL and SRIM were hospital practices, such as timely breastfeeding (BF) initiation, avoiding in‐hospital commercial milk formula supplementation, and BF counselling/support. By contrast, maternal overweight/obesity, caesarean section, and poor maternal physical and mental health were risk factors for DOL and SRIM. SRIM was associated with primiparity, the mother's interpretation of the baby's fussiness or crying, and low maternal BF self‐efficacy. Biomedical factors including epidural anaesthesia and prolonged stage II labour were associated with DOL. Thus, to protect against SRIM and DOL it is key to prevent unnecessary caesarean sections, implement the Baby‐Friendly Ten Steps at maternity facilities, and provide BF counselling that includes baby behaviours.

## Key messages

- Socioeconomic and demographic factors indicative of lower economic status increased the risk of self‐reported insufficient milk (SRIM) and delayed onset of lactation (DOL).
- Timely breastfeeding (BF) initiation and avoidance of in‐hospital commercial milk formula (CMF) supplementation are likely to reduce the prevalence of SRIM and DOL.
- BF counselling designed to prevent SRIM and DOL needs to strengthen maternal BF self‐efficacy, maternal understanding of baby behaviours such as fussiness and maternal wellbeing.
- Research is needed to better understand how the risk of DOL and SRIM increases with primiparity, caesarean sections, maternal overweight/obesity, and poor overall maternal health.
- Intervention studies specifically designed to reduce the risk of SRIM and DOL are urgently needed in low‐ and middle‐income countries.

## Graphical abstract

Modifiable protective factors identified through this systematic review for delayed onset of lactation (DOL) and self‐reported insufficient milk (SRIM) were hospital practices, such as timely breastfeeding (BF) initiation, avoiding in‐hospital commercial milk formula supplementation, and BF counseling/support. By contrast, maternal overweight/obesity, caesarean section, and poor maternal physical and mental health were risk factors for both DOL and SRIM.

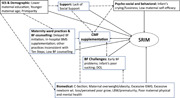

## 1 INTRODUCTION

The World Health Organization's (WHO) recommendation of exclusive breastfeeding (EBF) during the first 6 months, the introduction of complementary foods at this age, and breastfeeding (BF) continuation for at least the first 2 years of life continues to be supported by scientific evidence (Bartick et al., 2017; Chowdhury et al., 2015; Li et al., 2022; Pérez‐Escamilla et al., 2019; Victora et al., 2016). The fact that only 48% of children under 6 months old living in low‐income and middle‐income countries are exclusively breastfed and less than 70% of infants in low‐ and middle‐income countries are breastfed during their second year of life, with some regions having less than half of infants continuing BF beyond 1 year of age (UNICEF, 2022) is of public health concern. This is because of the numerous well‐known health, nutrition, and cognitive benefits that BF provides to children and women (Tschiderer et al., 2022; Victora et al., 2016). It has been estimated that over 800,000 annual deaths can be prevented among children under 5 years old by following this EBF recommendation (Rollins et al., 2016; Victora et al., 2016; Walters et al., 2016). This benefit also applies to high‐income countries; for example, a recent study using national data found BF initiation associated with a lower risk of post‐perinatal infant deaths across racial groups within the US population (Li et al., 2022). In addition, BF is friendly to the environment as most CMFs are made from cow's milk, use enormous amounts of water in their manufacturing, and leave large quantities of nonbiodegradable waste behind (Joffe et al., 2019; Smith, 2019).

Although several countries have been able to improve BF outcomes over the past two decades (Bhattacharjee et al., 2021; Neves et al., 2021), these improvements are not happening fast enough to achieve the 70% EBF goal by 2030 set by the Global Breastfeeding Collective led by WHO and UNICEF (Bhattacharjee et al., 2021). Hence, it is key to further understand how to address these breastfeeding challenges since birth.

For decades, BF problems have been commonly cited as one reason for early BF discontinuation, with the mother's complaint of not having enough milk, herein referred to as self‐reported insufficient milk (SRIM), being identified as the most common problem for not initiating or stopping BF. Indeed, SRIM is a major public health concern. It is frequently reported from the neonatal period and remains the most frequently cited reason by women all over the world for introducing commercial milk formulas (CMF, oftentimes misleadingly referred to as breast milk substitutes) (Gatti, 2008; Hill & Humenick, 1989; Huang et al., 2021). CMF introduction, in turn, is a strong risk factor for shorter EBF and BF durations (Pérez‐Escamilla et al., 2019; Segura‐Millán et al., 1994).

There have been several attempts to define and identify factors influencing SRIM. In 1979, Butz (1979) claimed that SRIM, referring to it as ‘Insufficient Milk Syndrome’, was simply a culturally acceptable reason for stopping breastfeeding and thus a socially acceptable excuse (Butz, 1979). Gussler and Briesemeister disputed this as the sole explanation, describing mother's insufficient milk as a ‘transcultural phenomenon’ since it affected mothers of different cultures and backgrounds (Gussler & Briesemeister, 1980). They also noted that SRIM was documented even among mothers motivated to BF, and among those with both good or poor nutritional status. They proposed that modernisation and urbanisation disrupted traditional feeding patterns through the separation of mother and child, which was not conducive to supporting breastmilk production and led to mother's interpretation of real or perceived insufficient milk. Thus, ‘Syndrome’ was described as ‘characterized by the lack of “constant contact” between mother and infant in modern urban settings' (Gussler & Briesemeister, 1980). The following year posited that decreased sucking stimulation of the nipples as a result of supplemental feedings was the most likely explanation for SRIM (Greiner et al., 1981).

In 1985, researchers agreed that SRIM was unlikely to be explained by a single factor and conceptualised SRIM as an outcome determined by a complex combination of factors, including maternal–child biological factors, sociocultural factors, health care practices and breastfeeding knowledge (Tully & Dewey, 1985). Consistent with this comprehensive approach to SRIM, findings from a literature review were used to propose an insufficient milk supply conceptual framework based on multifactorial determinants and mediators of milk production (Hill & Humenick, 1989). The multifactorial determinants fell into four categories (maternal time constraints, sociocultural factors, maternal comfort factors and infant factors) and the mediating factors included three categories (breastfeeding behaviour, maternal psychologic factors, maternal physiological factors). SRIM has been conceptualised as ‘a state in which a mother has or perceives that she has an inadequate supply of breastmilk to either satisfy her infant's hunger and/or support the infant's adequate weight gain’ (Hill & Humenick, 1989). Consistent with prior studies (Hill & Humenick, 1989; Huang et al., 2021; Mohebati et al., 2021; Segura‐Millán et al., 1994; Tully & Dewey, 1985), a subsequent SRIM literature review (Gatti, 2008) found that mother's self‐assessment of her milk supply was often associated with her perception of infant satiety or satisfaction mainly based on her interpretation of infant behaviours, especially crying or fussiness.

As mentioned above, while some researchers have suggested that SRIM is a sociocultural phenomenon, others have interpreted it as having physiological or biological causes or argued that it needs to be understood from a biocultural and behavioural perspective (Hill & Humenick, 1989). Yet, to date there has not been any systematic global analysis of SRIM risk factors. Hence, there is a need to systematically review the multiple factors contributing to SRIM, understand how they map across different socioeconomic, demographic, bicultural, psychobehavioural and health care systems domains, and identify pragmatic recommendations on how to address those factors that are modifiable.

Building on previous frameworks and empirical evidence (Chapman & Pérez‐Escamilla, 1999b; Dewey et al., 2003; Matias et al., 2010; Nommsen‐Rivers et al., 2010; Segura‐Millán et al., 1994), our research team recently suggested that in many instances, SRIM starts very early as a result of lack of information on what to expect during the colostrum phase or actually delayed onset of lactation (DOL), defined as milk ‘coming in >72 h post‐partum’ (Chapman & Pérez‐Escamilla, 1999b, 2000). The introduction of CMF products can then delay the onset of lactation even further, interfering with the establishment of the milk supply and increasing the risk of SRIM (Pérez‐Escamilla et al., 2019). Furthermore, others argue that the lack of access to qualified lactation counselling and stress management skills during the first days after birth, together with lack of knowledge among caregivers and/or family members on infant behaviours such as crying, push women into a vicious cycle that can lead to actual insufficient milk production (Karall et al., 2015). Researchers have acknowledged that maternal obesity should now also be considered a risk factor for DOL, SRIM, and shorter breastfeeding duration. This is because of consistent epidemiological evidence and strong biological plausibility indicating that maternal obesity can disrupt human lactation as a result of endocrinological alterations, mechanical barriers (large breasts preventing effective infant latch) and/or psychoemotional challenges such as low self‐esteem (Amir & Donath, 2007; Chang et al., 2020; Chapman et al., 2013; Pérez‐Escamilla et al., 2019). In short, DOL is a special case of SRIM as it happens during the period of time before ample milk secretion begins, known as stage II lactogenesis or lactation secretory activation stage (Boss et al., 2018). DOL is of concern because it has been associated with shorter EBF and BF durations (Chapman & Pérez‐Escamilla, 1999a; Huang et al., 2020).

To date, there are no reviews that have comprehensively synthesised the vast literature on DOL and SRIM. The main overall aim of this review is to increase the understanding of factors affecting SRIM and DOL to support the development and testing of interventions to improve BF exclusivity and duration. Thus, the objective of this systematic review is to answer the following questions:

(1) Which socioeconomic, demographic, and/or cultural factors increase the risk for SRIM, including DOL.
(2) Which behavioural and biomedical factors increase the risk for SRIM and DOL.

## 2 METHODS

The study protocol was developed and registered a priori in PROSPERO (ID#CDR42021240413). This review focus on studies with mothers and infants with no serious conditions that impede BF. The two main outcomes for the review were: (1) SRIM, defined as maternal report of not having ‘enough’ or ‘sufficient’ milk (e.g., not producing enough milk, milk dried up, baby hungry after feeding, not enough to satisfy the needs of the infant or DOL), as a reason for not initiating BF, stopping BF or introducing CMF; and (2) DOL, defined as perception of initiation of ample milk secretion beyond 72 h post‐partum. Review findings were reported following the preferred reporting systematic review and meta‐analysis protocols (Page et al., 2021).

### 2.1 Inclusion and exclusion criteria

Studies reporting SRIM or DOL were included only if they met the following criteria: (a) absence of serious maternal complications due to childbirth that might impede a timely initiation of breastfeeding such as severe post‐partum haemorrhaging; (b) post‐partum women delivering a singleton full‐term healthy baby infant or with no more than 10% of data coming from low‐birthweight or pre‐term infants; (c) quantitative studies with no design restrictions but with a comparison group or exposure; (d) only studies in English, Spanish or Portuguese; (e) studies conducted in high‐, middle‐ or low‐income countries; and (f) analysis of the association between mothers reporting DOL or SRIM during the first 6 months post‐partum and one or more of the following type of risk factors: sociocultural, economic, behavioural, knowledge or biomedical. Studies were excluded from the systematic review if they were qualitative studies, reviews, systematic reviews, meta‐analyses or quantitative studies with no comparison group. Studies that focused only on pre‐mature or low‐birthweight infants, included mothers or infants with conditions that might preclude breastfeeding, or were in a language outside of those mentioned in the inclusion criteria were also excluded.

### 2.2 Search strategy

We used a comprehensive search strategy developed by a medical librarian (K.N.), tested against validation articles previously identified by the authors, using both controlled vocabulary and free‐text queries. An independent medical librarian peer‐reviewed our electronic strategy using the Peer Review Electronic Search Strategies (PRESS) guidelines (McGowan et al., 2016). To prevent the omission of relevant studies, we used backward citation chaining, which involved reviewing the reference lists of articles identified and those from relevant literature reviews.

An initial exploratory search was conducted using a list of terms under the following concepts: (a) reasons for weaning or mixed feeding, or (b) SRIM (e.g., not enough milk, milk dried up, baby not full) or (c) DOL. The full search was conducted in the following databases in April 2021: MEDLINE ALL (via Ovid), Web of Science Core Collection (as licensed at Yale University, including SCI‐EXPANDED 1900‐, SSCI 1900‐, A&HCI 1975‐, CPCI‐S 1991‐, CPCI‐SSH 1991‐, BKCI‐S 2005‐, BKCI‐SSH 2005‐, ESCI 2015‐, and CCR‐EXPANDED 1985‐), PsycINFO (via Ovid), EMBASE (via Ovid), the Virtual Health Library Regional Portal (including LILACS), Scielo and Global Index Medicus. There were no time limits specified for this search. The complete final search strategy for MEDLINE is presented in Table 1, and all the searches are available at https://osf.io/jkx6s/.

**Table 1:** Search strategy: Ovid MEDLINE(R) ALL <1946 to April 16, 2021>

1	[SRIM SR search medline 2021‐04‐19]	0
2	(insufficient adj1 (breastmilk or breast milk or milk)).mp.	273
3	((milk or breastmilk) adj3 dried up).mp.	2
4	((milk or breastmilk) adj3 dry up).mp.	3
5	((baby or babies or infant* or newborn*) adj3 (hungry or "not full")).mp.	33
6	(reason* adj5 (mixed feeding or wean*)).mp.	152
7	("not enough milk").mp.	16
8	("not enough breastmilk").mp.	0
9	((breast milk or breastmilk) adj5 (early or low or insufficient or sufficient or inadequate or adequate or problems or perceived or perception or volume or supply or production)).mp.	1646
10	(breastfe* or breast fe* or infant feeding).mp. or exp Infant Nutritional Physiological Phenomena/	82,319
11	(milk adj5 (early or low or insufficient or sufficient or inadequate or adequate or problems or perceived or perception or volume or supply or production)).mp.	19,709
12	2 or 3 or 4 or 5 or 6 or 7 or 8 or 9 or (10 and 11)	4422
13	Milk, Human/	19,934
14	exp Lactation Disorders/or exp Lactation/	46,401
15	exp infant nutritional physiological phenomena/	60,528
16	(13 or 14) and 15	12,453
17	16 and (early or low or insufficient or sufficient or inadequate or adequate or problems or perceived or perception or volume or supply or production).ti,kf.	1309
18	12 or 17	5188
19	18 not (animals not humans).sh.	4718

#### 2.2.1 Study selection process and data extraction

**Figure 1:**
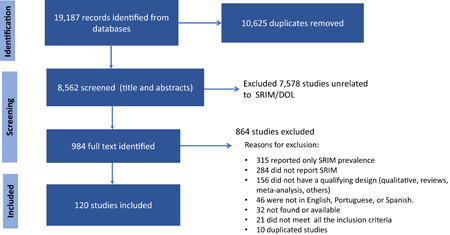
Self‐reported insufficient milk (SRIM)/delayed onset of lactation (DOL) systematic review. Preferred reporting items for systematic review and meta‐analysis flow chart

Covidence online software was used to conduct the screening process. Two of the researchers independently screened the first 200 titles and abstracts and compared their inclusion or exclusion assessments. Differences were resolved through a consensus process facilitated by the senior author (R. P. E.). Following this, three researchers (S. S. P., M. A., and R. P. E.) proceeded with independently reviewing the remaining titles and abstracts of each publication in Covidence (*n* = 8562), identifying 984 studies for full‐text review that were reviewed by all three authors. Discrepancies were resolved until consensus was reached among authors on the final list of included articles (Figure 1). Two researchers (S. S. P. and R. P. E.) extracted the following data from the included articles: study design, main outcomes, population and setting, main independent variable, other control variables, type of analysis, key findings and information required for quality assessment.

#### 2.2.2 Quality assessment and risk of bias

The Joanna Briggs Institute (JBI) critical appraisal tools appropriate for different study designs were used to assess the quality of observational and experimental studies (Munn et al., 2014). While JBI endorses the GRADE approach for systematic reviews and has similar approaches to assessing risk of bias, it has developed a wider variety of critical appraisal checklists for different research designs. Specifically, the JBI checklist for cross‐sectional studies is one of the newest and preferred tools for assessing the quality of evidence in systematic reviews. The critical appraisal checklists use a binary scoring process (i.e., yes/no) to assess quality, which can graphically display assessments of the methodological strengths and weakness of the literature, and it can also be transformed into scores assessing the evidence of the reviewed studies in a similar manner to the GRADE.

## 3 RESULTS

### 3.1 Search outcomes

Our search in seven databases identified 19,187 records, with no additional articles obtained from manual search of references and websites. After removing 6316 duplicates via the Yale deduplicator and 4309 duplicates in Covidence, 8562 records remained for screening. After reading through titles and abstracts we identified 7578 articles that were not related to SRIM or DOL leaving 984 articles meeting the eligibility criteria for full‐text review. A total of 126 studies were initially identified as meeting the inclusion criteria and six more articles were excluded during extraction; three articles were excluded due to the design not qualifying and three were excluded because they only reported SRIM prevalence. Finally, 120 studies qualified for inclusion for this review (Figure 1).

### 3.2 Study characteristics

A total of 37 (31%) studies were conducted in two countries from North America, followed by 26 (21.6%) studies conducted in 10 countries from Europe and Central Asia region; 25 (21%) studies conducted in seven countries from the East Asia and Pacific region; 15 (12.5%) studies conducted in eight countries from Latin America and the Caribbean; 7 (6%) studies conducted in three countries from the Middle East and North Africa; 5 (4%) studies conducted in four countries from South Asia; 3 (2.5%) studies conducted in three countries in Sub‐Saharan Africa; and 2 (1.7%) included multiple countries and regions. While 79 studies were conducted in high‐income countries, 30 were conducted in upper‐middle‐income, 10 in low‐middle‐income countries, and 1 in both a high income and an upper middle country. With respect to study design, 33 studies were cross‐sectional, 33 were prospective and 25 descriptive (i.e., did not conduct formal statistical analyses) (12 cross‐sectional, 6 prospective, 5 retrospective and 2 quasi‐experimental), 12 were retrospective, 10 were randomised controlled trials (RCTs), and 7 were quasi‐experimental. Of the SRIM studies reviewed, 10 were published between 1979 and 1989; 21 from 1990 to 1999; 24 from 2000 to 2009; 33 from 2010 to 2019 and 10 from 2020 to 2021. Six DOL studies were published from 2000 to 2009, 10 from 2010 to 2019 and 2 in 2020. The four studies assessing both DOL and SRIM data were published between 2009 and 2021.

Of the 120 studies, 98 focused on SRIM, 18 on DOL, and 4 on both outcomes. Of the SRIM studies, 19 used a scale yielding a self‐reported maternal milk sufficiency score (Table 2) and the rest obtained SRIM as a direct response for not initiating BF (*n* = 8) or for stopping breastfeeding (*n* = 52), starting infant CMF (*n* = 30), introducing other liquids or solids (*n* = 9), or as part of a list of maternal BF problems or concerns (*n* = 33). Among these SRIM studies, it was not possible to tease out when SRIM referred to insufficient milk production, low milk quality, or both. Of the studies reporting maternal perception of onset of lactation, DOL was defined in different ways. Studies identified DOL (>72 h post‐partum) based on maternal self‐report of breast fullness symptoms (*N* = 8) and perception of when their milk came in (*N* = 6). The remaining DOL studies used a variety of approaches to determine maternal self‐report of DOL.

**Table 2:** Scales used to assess the maternal perception of her milk supply

Scale	Description	Studies	Comment
Perceived Insufficient Milk Questionnaire (McCarter‐Spaulding & Kearney, 2001)	6 items; first item asks if mother has enough milk to satisfy her infant (yes/no); 5 items include “My baby generally appears satisfied after feedings”; 10‐point Likert‐scale numeric (0 [strongly disagree]–10 [strongly agree]); higher score = better milk supply perception	Galipeau et al. (2017), Gokceoglu and Kucukoglu (2017), Menekse et al., (2021), Otsuka et al. (2008), Yilmaz et al. (2020)	Based on The Parent Expectation Survey to measure self‐efficacy in early parenting developed (Reece, 1992)
H&H scale (Hill & Humenick, 1996)	20 items addressing 3 constructs: maternal BF confidence/commitment; perceived infant satiety (5 items); maternal/infant breastfeeding satisfaction (5 items); 7‐point Likert‐scale (1 [strongly disagree]–7 [strongly agree]); higher score = better milk supply perception	Huang, Gau, et al. (2009), Huang, Lee, et al. (2009), Lin et al. (2011), Samuel et al. (2012)	Based on insufficient milk syndrome framework (Hill & Humenick, 1989)
SRIM problems score (Henly et al., 1995)	Mother's rating of the adequacy of her milk supply; constructs: insufficient letdown, not enough milk, baby weight gain, frequency of nursing, and baby crying or dissatisfied; 7 point Likert‐scale (0 [no problem] to 7 [major problem]); higher score = more insufficient milk perception	Duckett et al. (1998)	
BF perception questionnaire	5 items; 1 on mother's level of BF confidence (1 [strongly agree]–4 [strongly disagree]), 4 rating mother's perception of: infant feeding frequency (not often enough, normal, too often); amount of baby takes each time (too little, normal, too much); time infant takes at each feed (too slow, normal, too fast); mother's perception of her milk production (too low, normal, too high)	Kent et al. (2015)	BF perception questionnaire
Insufficient milk perception	SRIM as a BF concern, assessed with 6‐point Likert‐scale (1 [not at all adequate]–6 [adequate]). Items: ‘To what extent do you think you have adequate milk supply to breastfeed your baby?’, ‘To what extent do you think your breasts are able to produce an adequate amount of milk for your baby?’, ‘To what extent do you think your baby's crying is related to the adequacy of your milk supply to breastfeed your baby?’	Wood et al. (2017)	Scale validated in this study
BF Adaptation Scale (BFAS) (Sun‐Hee, 2019)	16 items and 6 BF subdimensions emotional exchange with one's baby, BF confidence, sufficient breast milk, baby's feeding capability, baby's satisfaction with breastfeeding, and maintenance of breast milk volume; each item rated from 1 (disagree)–5 (absolutely agree); higher score indicates better BF adaptation; sufficient milk subdimension includes 3 items	Sun‐Hee (2019)	Validation of a shorter version of the BFAS; adapted form 27‐item and 8 BF subdimensions of original BFAS
Breast Milk Perception Scale for Adults (Eren, 2016)	30 items; 5 point Likert‐scale (1 [strongly agree]–5 [strongly disagree]); higher score = better milk supply perception	Donmez and Korgali (2021)	This scale is not available in English
BF Perception Questionnaire 2	14 items; 5 Likert‐scale (strongly agree to strongly disagree); Q2–5 addressed growing well, wet nappies, soiled nappies, baby alertness; Q6–10 addressed suckling, baby satisfaction with feeds, frequency and length of feedings; Q11 addressed need of supplementary formula; Q12–14 address fullness of breast*	Kent et al. (2021)	SRIM if participants answered unsure, disagree or strongly to ‘I think I produce enough breast milk for my baby’
Statements for mother's decision to wean their infant	SRIM report based on 5 statements:‘Breast milk alone did not satisfy baby’; ‘Baby was not gaining enough weight’; ‘A health professional said baby was not gaining enough weight’; ‘Mom had trouble getting the milk flow to start’, ‘Mom did not have enough milk; 4 point Likert‐scale’ (1 [Not important at all]–4 [very important])	Whipps and Demirci (2021)	Responding 3 or 4 to Likert scale for reach item indicative of SRIM

The overall characteristics of the included studies, including SRIM and DOL prevalence reported, are summarised in Tables 3 and 4 and detailed information including statistical coefficients of associations can be found as online Supporting Information Materials (Appendix SA). As expected, both SRIM and DOL were highly prevalent worldwide (Tables 3 and 4). On average, SRIM was reported by 44.8% of women for introducing CMF (range: 10.5%–73.1%) and 33.8% of women for stopping breastfeeding (10.0%–74.2%) (Table 3). Furthermore, 28.9% of women reported DOL (Table 4).

**Table 3:** Self‐reported insufficient milk study characteristics by study design

Author (year), country	Population/setting	SRIM risk factors	SRIM prevalence
*Descriptive cross‐sectional* (*n* = 12)			
Amine et al. (1989), Kuwait	Mothers of children <3 years Community infant feeding practices survey (*N* = 2436)	Younger mothers (<35 years)	30.7% reason for weaning
Chuang et al. (2007), Taiwan	1783 mothers Taiwan National Birth Registration database Home interview at 6 mo pp	Highest among foreign‐born unemployed women Similar among all employed women Taiwanese unemployed women had lowest prevalence	52% (45.7%–65.9%) not ever BF
Florack et al. (1984), Leiden, the Netherlands	Mothers of 4‐mo‐old (*N* = 189) and 6‐mo‐old infants (*n* = 151) Recruited at Child Health Units Infant feeding practices questionnaire at home	Similar SRIM prevalence as a reason for bottle feeding: (*n* = 71) at 4 mo (47%) and 6 mo (41%)	41%–47% at 4–6 mo pp Reason for bottle feeding
Heldenberg et al. (1993), Hadera County, Israel	Women with 6 mo old infants (*N* = 876) Well baby clinic	SRIM for not BF > 4 wks: Jewish (40.8%) versus Arab (13.3%) women SRIM for stopping BF: Jewish (47.2%) versus Arab women (57%)	13.3%–49.8% Not BF 47.2%–57% Formula introduction
Meirelles Cde et al. (2008), Rio de Janeiro, Brazil	Mothers introducing formula while rooming‐in with their newborns at a BFH (*N* = 300)	SRIM: 20% <1 h, 49% 1–12 h pp, 43% 12–96 h pp	37.3% In‐Hospital formula
Mizuno et al. (2004), Tokyo, Japan	Mothers with 6–12 mo infants (*N* = 1474)	Infant sucking behaviours SRIM was the most frequent reason for stopping BF in the ‘barracuda’ (27.4%), ‘procrastinators’ (32.7%), ‘resters’ (47.6%)	38.2% Stopping BF
Moran Rey (1992), National Survey, Spain	A national sample of mothers with 3–18 mo old children (*N* = 1061)	No clear patterns between SRIM and mother's age, parity, and city size –Lack of milk Mother's age: >32 (37.1%) versus younger (<25 [31.1%]; 26–28 [31.9%]; 29–32 [30.7%]) –Baby hungry Mothers' age: <25 (43.3%) hungry versus older mothers (26–28 [37.2%], 29–32 years old [41.1%]; >32 [31.7%]) –Lack of milk by parity: Primiparous (31.1%); multiparous (34%) –Baby hungry Primiparous (39.7%); multiparous (37%) –SRIM by city size: >400,000 inhabitants (26.1%); the smaller cities 5000–30,000 (33.3%), 30,000–400,000 (32%) –Baby hungry: >400,000 (40.1%); 30,000–400,000 (40.0%); versus 5000–30,000 (34.6%)	74.2% Stopping BF
Oommen et al. (2009), New Delhi and Haryana, India	Urban and rural mothers; newborns >1500 g, gestational age >34 weeks (*N* = 284); assessed 6 mo pp	Area of residence Non‐EBF by 52% of urban and 50% rural mothers during hospital stay due to perceived insufficiency of milk	50%–52% in hospital 36%–47% first 3.5 mo 37%–62% 3.5–6 mo pp Not EBF
Perez‐Escamilla et al. (1997), Mexico City, Mexico	Mothers delivering at two maternity wards with rooming‐in (*N* = 518), and a C‐section ward with no rooming‐in (*N* = 247)	SRIM at 4 mo: rooming‐in (31%), partial rooming‐in (46%), not rooming‐in (32%)	31%–46% Stopping BF
Tai‐Keun and Berlin (1981), Rural region, Korea	Participants with children ≤30 months old living in seven marginal rural areas (*N* = 337 participants that introduced formula)	SRIM by birth attendant, midwife (61.3%); physician (69%); neighbour/friend (23.9%); family (32%)	65.6% mothers formula introduction
West (1980), Edinburgh, Scotland	Mothers with full‐term babies; mailed questionnaire at 6 mo pp (*N* = 116)	SRIM by infant age: 57%, <6 wks; 6–11 wks, 46.1%; between old (*n* = 39); 12–22 wks, 42.8%	50% Stop BF
Zurayk and Shedid (1981), Nabatieh and Sidon, Lebanon	BF urban and rural women (*N* = 253)	SRIM: 66.7%, rural women; 67.3%, urban women	67.3%–66.7% Stop BF
*Descriptive prospective* (*n* = 6)			
Colin and Scott (2002), Perth, Australia	BF women filling‐out a Self‐administered questionnaire at 2 hospitals (*n* = 490) Phone survey at 2, 6, 10, 14, 18, and 24 wks pp	No clear pattern as a function of infant age	23% BF problem at the hospital 16.7% reason for introducing infant formula after discharge
Essex et al. (1995), National Child Health Data, New Zealand	Infants <6 mo (*N* = 3929) European, Maori and Pacific Islanders National Child Health Study Assessments at birth to 6 wks, >6 wks to 3 mo, >3 to 6 mo pp	No clear pattern as a function of infant age	29%–33% Reason for stopping BF: Birth to 6 wks: (29%); >6 wks to 3 mo (29%); >3–6 mo (33%)
Graffy (1992), Nottingham, UK	Pregnant women (*N* = 514) Assessed at 25 wks gestation, 6 wks and 6 mo pp	87% related SRIM to infant being unsettled; 11% to feeling breasts less firm and 10% to poor infant's weight gain	46% mentioned as BF problem. 47% as reason for stopping BF at <6 wks and 59% at >6 wks
Perez‐Escamilla et al. (1993), Hermosillo, Sonora, México	Women planning to BF; vaginal delivery, healthy term infant (*N* = 165). Women interviewed in the hospital, 1 wk, 2 mo, 4 mo pp	SRIM as reason for giving formula: Hospital (50%); 1 wk (63%); 2 mo (76%); 4 mo (74%) (34/46) SRIM as a reason for stopping BF: 2 mo milk (40%); 4 mo (41%)	50%–76% Formula introduction 40%–41% Stopping BF
Schwartz et al. (2002), Detroit, Michigan and Omaha, NE, USA	BF women (*N* = 1422); recruitment: birth centre (Detroit), work site (Omaha)	SRIM as a reason for stopping BF 37.6% at 3; 35% at 6; 25% at 9; 13.9% at 12 wks pp	13.9%–35% Reason for stopping BF
Sun et al. (2017) Guangzhou, China	BF mothers; full‐term healthy babies (*N* = 180 stopping BF)	SRIM: 38.5% at 1 mo; 56.7% at 2–4 mo; 52.4% at 5–6 mo pp	38.5%–52.4% Reason for stopping BF
*Descriptive retrospective* (*n* = 4)			
Andrade Barcia and Valle Carrera (1981), Manabí Province, Ecuador	962 women from 2 cities Child ≤3 years old Community infant feeding practices survey	Younger infant age	20%–34% not ever BF 20%–22% Stopping BF
Keemer (2013), Brisbane, Australia	BF women giving birth to a singleton healthy, full‐term infant at a birthing facility (*N* = 128) Infant feeding questionnaires mailed during first 7 days pp	Primiparity Primiparous (69.5%) reported SRIM more often than multiparous (34.4%) women	37% BF challenge
McCann and Bender (2006), Cochabamba and Santa Cruz, Bolivia	Infant feeding patterns survey conducted in 1991 in Cochamba (*N* = 400), and in 1994 in Santa Cruz (*N* = 434) with children <18 mo old	SRIM less prevalent in Cochamba versus Santa Cruz Infant's crying most common SRIM cue	15.6%–24.5% Stopping BF 7.8%–19.8% Fluids introduction
Mosha et al. (1998), Morogoro District, Tanzania	Rural and urban mothers of ≤2 years old children (*N* = 400)	SRIM as reason for introducing weaning foods more common in rural (73%) versus urban (41.5%) areas	41.5%–73% Solid foods introduction
Yang et al. (2004), Alberta, Canada	1996–1997 National Population Health Survey (*N* = 1113, 949 initiated BF)	SRIM: <1 wk, 18.5%; 1–12 wks, 29.4%; 3–6 mo, 17.3%; ≥7 mo; 2.1%	2.1%–29.4% Stopping BF
*Descriptive quasi‐experimental* (*n* = 2)			
Houston et al. (1981), Edinburgh, Scotland	BF women delivering a full‐term (*N* = 80) –IG: home visitors plus midwife visit and telephone number for BF support –CG: only health visitors	Lack of BF support SRIM at 24 wks pp: 19% in CG gave SRIM as a reason to stop BF versus none of the mothers in IG	19% Stopping BF
Whichelow (1979), Cambridge, England	Mothers from 2 geographical areas; hospital recruitment; At 2 wks pp women in one areas given dietary advice to produce milk	Less mothers in the dietary education group reported SRIM; 13% versus 24% in comparison group	31.5% Stopping BF by 2 wks 13%–24% Introducing Formula between 2 and 3 mo
*Cross‐sectional* (*n* = 27)			
Bryant et al. (2019), Internet Survey, USA	BF women (≥18) with 3–9 mo old infants Hormonal contraceptives users (*N* = 852) Not using hormonal contraceptives (*N* = 2070) internet‐based survey	NS between group SRIM prevalence difference	41% BF concern
Einterz and Bates (1994), Northern Cameroon, Cameroon	Mothers with children <2 years (*N* = 534) Recruited at a health centre	SRIM among mothers of children <5 mo: non‐Muslim versus Muslim (17% vs. 51%) Colostrum cultural beliefs seem to prevent early BF initiation in this population and a higher perception of SRIM	17%–51% BF problem
Fawzia and Ezzat (1997) National data, Kuwait	Mothers of children <2 years old Interviewed at home	Maternal illiteracy SRIM higher among illiterate (67.5%) versus secondary (44.8%) and university graduates (38.9%)	53.1% Reason for introducing formula 36.1% Reason to stopping BF
Gokceoglu and Kucukoglu (2017), Eastern, Turkey	BF >18 years‐old mothers (*N* = 200) with full term newborns	Lower BF self‐efficacy; younger women, <university degree, lower income, female infant	Not available
Guelinckx et al. (2012), Leuven, Belgium	Women selected in maternity hospital (*N* = 200) Based pre‐pregnancy BMI from medical chart Telephone BF survey at 3 and 6 mo pp	Maternal underweight and obesity SRIM by BMI: Underweight (26%); Normal weight (13%) Overweight (18%); Obese (24%); *p* = 0.041.	19% Reason for stopping BF
Gumussoy et al. (2020), Western Anatolia, Turkey	351 Literate women (*N* = 351) Hospital delivered full‐term infant Attending child vaccination clinic at 4–8 wks pp	Lower breastfeeding self‐efficacy Lower maternal attachment	8.6% Based on scale
Henly et al. (1995), North Central, USA	BF primiparous women with full‐term infants (*N* = 620) Maternity hospital	Maternal anaemia SRIM: Women with (19.7%) versus without anaemia (11.4%), *p* = 0.01	13.2% BF problem 20.8%–29.6% Weaning reason
Hazrat et al. (2017), Quetta, Pakistan	BF women (*N* = 100, 17–45 year old) with ≤3 mo old infants Immunisations clinic	Maternal depression SRIM: 44% with versus 13.5% without depression (*p* < 0.001)	26% Direct SRIM question to mothers
Hill and Aldag (1991), Midwest, USA	Women initiating BF within 24 h pp (*N* = 384) Delivery of single infant Recruited between 8.1 and 14 wks pp Filled questionnaire at paediatrician's or WIC office	Baby behaviour (fuzzy, refused breast, poor feeder) and poor weight gain low maternal BF confidence; low partner support, poor mother's health, mother‐in‐law BF disapproval, and low birth weight	26% Reason for not satisfying baby's hunger
Hill et al. (1997), Midwest and Wyoming, USA	*N* = 51 women from 5 hospitals with prior or no prior BF experience	No significant SRIM differences between first (33.3%) and second (22.2%) time breastfeeding women	30.30% Stopping BF
Huang, Lee, et al. (2009) Northern Taiwan	Healthy mother and their full‐term infants (*N* = 205); BFH	Mixed feeding with formula; lower planned BF duration; suboptimal infant sucking; no BF family support these factors explained 35% of variance in H&H scale score	Not available only H&H scales mean score provided
Hurley et al. (2008), Maryland, USA	WIC participants with children <12 mo (*N* = 323)	Hispanic ethnicity SRIM more likely among Hispanic (41.3%) than African American (19.5%), White (18.4%) women, *p* = 0.001	23.4% BF cessation
Jarlenski et al. (2014), National survey, USA	Nonobese and obese mothers of heathy term newborns (*N* = 2997)	Maternal obesity obese more likely than nonobese women to report SRIM for stopping BF	23%–26% Not ever BF 45%–51.3% Stopping BF < 6 mo
Kair and Colaizy (2016) PRAMS survey Illinois, Maine and Vermont, USA	Women who had initiated and discontinued BF data from Pregnancy Risk Assessment Monitoring System (PRAMS) (*N* = 6467)	Maternal overweight/obesity More SRIM among overweight (OR: 1.39, 95% CI: 1.16–1.68) and obese (1.26, 1.03–1.54) than normal‐weight women	42% Stopping BF early
Kim et al. (2013), South Korea, Korea	Korean women recruited at drug stores, shopping malls, and street corners in urban and rural areas in (*N* = 516)	Pigs' feet consumption SIM reported by 37% of mothers consuming pig feet versus 29% among nonconsumers, *p* < 0.001.	23%–65% Stopping BF
Lin et al. (2011), Northern Taiwan	BF women; planned C‐section; delivering full‐term infant (*N* = 141); data collected during 3 d pp	Delayed BF initiation, early neonatal formula introduction, not BF during first 72 h pp; lower BF frequency; epidural (vs. spinal)	Not available H&H scale
McCarter‐Spaulding and Kearney (2001), Northern, USA	BF mothers with 1–11 wk sinfants 1–11 wks of age (*N* = 60); mailed questionnaire	Mother's parenting self‐efficacy associated with milk supply scale score (*r* = 0.487, *p *< 0.01)	Not available scale used
Menekse et al. (2021), Sakarya province, Turkey	BF mothers with healthy full‐term 0–2 mo old infant *N* = 429)	Lower maternal education; not BF within the first 24 h pp; not EBF; BF self‐efficacy	Not available scale used
Monteiro et al. (2011), Ribeirão Preto, Brazil	BF mothers with a healthy infant <4 mo; vaccination clinic (*N* = 231)	SRIM RF's: shorter duration of feedings; shorter intervals between feedings; child not satisfied after feeding SRIM cues: infant crying, frequent feeding	29% Not having good/enough milk
O'Sullivan et al. (2015), National Survey, USA	IFPS II mothers (*N* = 1731); 1 and 2 mo pp	Maternal obesity Indirect effect of obesity on EBF outcomes through SRIM among primiparous and multiparous women at both time points SRIM factor analysis: too long for milk to come in; baby did not gain/lost too much 20 weight; nipples were sore, cracked, or bleeding; mom did not have enough milk for the baby; baby had trouble sucking or latching; baby got distracted	8.9%–19.3% not enough milk 43.4%–51.1% trouble getting milk flow to start
Otsuka et al. (2008), Tokyo, Kusatsu, Kyoto, Japan	In‐hospital BF mothers delivering a full‐term baby (*N* = 262); self‐completed survey at 1 mo pp	Breastfeeding self‐efficacy Higher in‐hospital BF self‐efficacy inversely associated with SRIM at 4 wks pp	73% Formula introduction
Robert et al. (2014), Wallonia and Brussels, Belgium	Immunisation surveys; 16 BF questions about BF in‐hospital and at discharge (*N* = 1069)	–SRIM RF's (stop EBF <5 mo and BF <6 mo): Wallonia: lower maternal and paternal education; lower paternal education, lack of awareness of WHO infant feeding recommendations; pre‐term baby; low maternal BF satisfaction –SRIM RF's (stop BF <6 mo): Brussels: lower maternal education, not delivering in a BFH (*p* = 0.08), low BF satisfaction	20.7%–23.8% Stopping EBF <5 mo 23.8%–25% Stopping BF <6 mo
Rodrigo et al. (2019), Ragama, Sri Lanka	BF Mothers recruited >24 h to <7 d pp (*N* = 249); in‐hospital self‐administered questionnaire	SRIM RF's: family member telling mother she had low milk supply; <30 years; C‐section; antenatal maternal complications; less education; low infant's urine output; Post‐natal SRIM factors: staff telling mother she had inadequate milk supply, not being a good mother	21% perceived their milk as inadequate.
Rozga et al. (2015), Michigan, USA	WIC eligible women receiving services from a BF peer counsellor (PC) programme (*N* = 7942); secondary analysis of women who discontinued breastfeeding while enroled in the PC programme	SRIM RF's: Hispanic; ≤5 PC versus ≥9 contacts, *p* < 0.001 SRIM report for stopping BF by infant age: 8.7% at <1 wk; 21.8% between 1– <4 wks; 25.6% between 4 and <3 mo; 19.8% between 3 and <6 mo; 15.1% between 6 and <12 mo	20.9% Reason for stopping BF
Sandhi et al. (2020). Yogyakarta City, Indonesia	Mothers; full‐term birth; filled out survey (*N* = 230)	No skin‐to‐skin contact; lower BF self‐efficacy	Not available Scale score
Tully and Dewey (1985), Davis, CA, USA and Kingston, Jamaica	Post‐partum mothers in CA (*n* = 114) and Jamaica (*n* = 92) interviewed at 6 wks pp	Perceiving BF as inconvenient; in‐hospital formula; low‐birth weight; Mexican‐born without in‐hospital BF education; multiparae; baby crying	27.2%–47.4% Formula introduction
Whichelow (1982), Cambridge, England	Women attending pre‐natal childbirth classes; maternity hospital supportive of BF (*N* = 130)	Not perceiving milk ‘let down’ associated with SRIM, *p* = 0.02	20.3% BF problems 10.0% Stopping BF
*Prospective* (*n* = 22)			
Brown et al. (2014), Nova Scotia, Canada	500 mothers who stopped BF before 6 mo Data from the Healthy Beginnings Public Health and Nova Scotia Atlee Perinatal Databases Assessments at <1 wk pp, 1–6 wks, >6 wks	Younger maternal age (<25 years); Primiparity; Living in high‐income neighbourhoods NS differences on SRIM report first 1–6 wks (23.2%) versus >6 wks pp (20.5%)	21.6% BF cessation <6 mo
Cooke et al. (2003), Sydney, Australia	Pregnant women (28–36 wks) *N* = 3 hospitals; *N* = 449 women Postal surveys antenatally and at 2, 6 wks, and 3 mo pp	Lower BF satisfaction scores at 2 wks pp Lower infant satisfaction score at 2 wks; 6 wks, and 3 mo pp	14% at 2 wks 17% at 6 wks 12% at 3 mo BF problem
Donmez and Korgali (2021), Sivas, Turkey	Parents with newborns (*N* = 332) Interviewed at the hospital and by phone at 2, 3, 6 mo pp	C‐section delivery Lack of maternal BF training	Reason for stopping BF 6.66% at 2 mo 32.50% at 4 mo 30.72% at 6 mo
Duckett et al. (1998), Midwestern, USA	BF primiparous women (*N* = 602) Full term delivery at a large urban private US hospital	5 socioeconomic, demographic and behavioural factors	Not available
Flaherman et al. (2016), Hershey, PA, USA	BF mothers with infants born ≥34 wks at two hospitals (*N* = 1107) Assessment at hospital and 2 wks pp	In‐hospital newborn excessive wt. loss (EWL) (≥10%) Maternal pp anxiety: 10.2% of women with SRIM had positive anxiety score (PAS) versus 6.1% w/o SRIM	20% (EWL)−40% (not EWL) at 2 wks pp Milk supply concern
Forman et al. (1992), Negev, Israel	Bedouin infants >2500 g at birth	Spring–summer delivery; 0–2 mo old (SRIM OR: 1.65, 95% CI [1.19–2.31]) Multiparity among 3–18 mo old (1.12, 1.04–1.22)	72% at 2 mo, 28% at 7–18 mo pp Reason to introduce the bottle
Galipeau et al. (2017), Canada	BF primiparous women ≥18 years with full term infant (*N* = 123) non‐Baby Friendly Hospital. SRIM assessed at 48 h and 2 wks pp	At 48 h pp: Poor suckle; infant irritability; lower BF self‐efficacy At 2 wks pp: lower BF self‐efficacy; lower number of BF/24 h No relationship with baby's wt loss at 48 h and actual 24‐h milk production at 2 wks pp	18% at 48 h pp 7.5% at 2 wks pp Direct SRIM question to mother
Herrera (2008), Carolina, Ecuador	Mothers delivering vaginally delivery of full term healthy infant (*N* = 47) infant Interviewed at home at baseline, monthly for 5 mo	Lowest and highest income SRIM: lowest (40.9%), highest (46.2%), versus middle (12.5%) income *p* = 0.043	12.5%–46.2% BF problem
Hillervik‐Lindquist (1992), Uppsala, Sweden	Women with healthy infants EBF at hospital discharge (*N* = 51)	–Less sexual desire at 3 mo pp a higher proportion of women with SRIM reported (81%) vs those W w/o SRIM (30%) (*p* < 0.001) –Primiparity Women with SRIM more likely to be primiparae (71% with vs. 48% without SRIM) –NS differences for infant's birthweight, delivery mode, timing of 1st BF; suckling durantion during the first 6 mo pp	55% BF problem
Hillervick‐Lindquist et al. (1991), Uppsala, Sweden	Women with healthy infants EBF at hospital discharge (*N* = 51). Women were asked to measure 24 h milk production (test‐weighing method).	Breastmilk production among EBF women reporting SRIM was significatively lower at 3 mo (*p* < 0.01) and 5 mo (*p* < 0.05) but adequate in both groups. The infants from women with SRIM also had a significantly lower weight‐for‐age (wt/age) at 2, 3, 4 and 9 mo, and were significantly thinner. Infants wt/age from both groups were above the NCHS mean.	55% BF problem
Hillervik‐Lindquist et al. (1991), Uppsala, Sweden	Women with healthy infants EBF at hospital discharge (*N* = 51) Monthly home visits up to 6 mo, then telephone contact up to 18 mo	Earlier start of gradual weaning process –SRIM mothers more likely to start weaning process gradually replacing breast milk feeds with formula/solids (57.1% vs. 39.1%) –Non‐SRIM more likely to offer solids between BF sessions	55% BF problem
Huang, Gau, et al. (2009), Northern Taiwan	Healthy mothers and full‐term infants (*N* = 205); BFH; Breast milk via BF (BG); Supplementary formula via cup (CG); Supplementary formula via bottle (BG)	Milk supply perception score significantly higher in the BG and CG groups versus BG at 3 d, 2 wks, 4 wks pp	Not available only scale scores provided
Karkee et al. (2014), Kaski District, Nepal	Women ≥5 mo pregnancy (*N* = 701)	Older infant age SRIM increased from 15% at 4 wks to 45.3% at 22 wks pp, *p* < 0.001	15%–45.3% Perception that breast milk was insufficient
McCann et al. (2007), National survey, USA	Mothers enroled in WIC since pregnancy; WIC IFPS (*N* = 874).	Infant age SRIM: 34% at 1 mo, 25% at 3 mo, 15% at 5 mo pp	15%–34% BF problem 55% Breast milk supply adequacy question
Mohebati et al. (2021), Mexico City, Mexico	Primiparous mothers planning to BF, and not working for at least 6 mo pp (*N* = 475) Assessed at BFH, 1, 4 wks pp	SRIM RF's More BF problems; baby crying more than expected, DOL	19% BF problem 63% Milk supply question
Mok et al. (2008) Poitiers, France	Women delivering a full‐term baby at University Hospital (*N* = 222) Assessments: Hospital, 1 mo, 3 mo pp	Maternal obesity Fewer obese versus nonobese mothers perceived adequate milk supply at 1 (60% vs. 94%) and 3 (55% vs. 92%) mo pp	33.3%–55.25% Stopping BF
Perez‐Escamilla et al. (1992), Hermosillo, México	Women with vaginal delivery of a healthy term infant at a rooming‐in (RI) and non‐RI (NRI) maternity ward (*N* = 73)	Non‐rooming‐in RI versus NRI SRIM: hospital (32%, 60%, *p* < 0.01), 8 d (47%, 39%), 16 d (35%, 43%); cue: baby crying	32%–60% Formula in hospital 35%–47% Stopping BF
Li et al. (2008), National Survey, USA	IFPS II survey BF mothers delivering a healthy singleton infant (*n* = 1323)	Hispanic; low‐income SRM higher among Hispanic (vs. White) mothers; lower income	45.5%–49.5% Stop BF
Sun‐Hee (2019), South Korea	Women recruited at post‐partum centres; interviewed at 2 and 4 wks pp (*N* = 461)	Employed, C‐section with lower sufficient breast milk, and baby's satisfaction with lower milk supply scores	Not available Scale score
Wagner et al. (2013), Davis, CA, USA	Primiparous women recruited prenatally (*N* = 418)	SRIM for formula: 0 d, 28%; 3 d, 42%; 7 d, 27%; 14 d, 19%; 30 d, 20%. Stop BF: 0 d, 26%; 3 d, 40%; 7 d, 26%; 14 d, 19%; 30 d, 20%	42% Formula introduction 40% Stopping BF
Wang et al. (2014), Hong Kong, China	Women; 5 hospitals; mailed questionnaires; pregnancy, 6 wks pp (*N* = 610)	SRIM by infant's age: <1 wk, 33.8%; 1–3 wks, 36.5%; 1–3 wks, >3–6 wks, 27.7%, *p* = 0.550	32.7% Stopping BF
Wood et al. (2017), Pacific Northwest, USA	BF mothers; full‐term baby, recruited at 48 h; SRIM reduction intervention; 3 home visits (*N* = 15)	The intervention significantly decreased attribution of infant crying to SRIM during first mo pp (*p* ≤ 0.001).	43% BF problem
*Quasi‐experimental* (*n* = 7)			
Kent et al. (2015), Western Australia	BF mothers between 2 and 13 wks pp, normal BW, full‐term infant (*n* = 203) BF experience questionnaire before and after two 24 h test‐weighing 4 wks apart	Not test weighing SRIM decreased from 39% before to 22.3% after weighing, *p* = 0.008 Low milk intake perception decreased from 49.5% to 31%, *p* = 0.009	41.8% Low milk production 54.18% Infant little breast milk consumption per feed
Kent et al. (2021), Perth, Australia	BF mothers (*N* = 387) BF perception questionnaire before and 2–4 wks after their second BF clinic consultation	SRIM RFs (*p* < 0.05) Primiparity; non‐Caucasian; C‐section; DOL; anaesthesia; using formula; perceiving infant slow weight gain	44% BF problem
Nommsen‐Rivers et al. (2009), Northern CA, USA	Primiparous low‐income mothers; healthy baby; doula (*n* = 44) versus standard of care (*n* = 97); hospital, 3 d, 6 wks pp	Lack of doula care Doula group more likely to not have milk concerns (71.8%) versus SoC (62.4%) at 3 d pp	28.2%–37.6% Breast milk concerns
Silbert‐Flagg et al. (2020), Northeast, USA	BF mothers; hospital BF support group; online survey (*N* = 100)	Pre: 22.9% not at all, 47.9% somewhat, 29.2% very concerned about milk supply. Post: 37.9%, 41.0%, 21.0%; *p* < 0.01	62%–77% Milk production concern
Ume et al. (2014), Rawalpindi, Pakistan	BF multiparae; with or without pre‐natal BF support (*N* = 100)	SRIM: control (40%); intervention (16%), *p* < 0.008	16%–40% BF problem
Vázquez Cancela et al. (2018), Santiago de Compostela, Spain	BF mothers; standard of care (CG, *N* = 54); plus BF education (IG, *N* = 56) before discharge	SRIM RF's 1 mo: CG‐30.8%; IG‐69.2%, *p* = 0.06 4 mo: CG‐50%; IG‐50%	30.8%–69.2% Stopping BF
Yilmaz et al. (2020), Eastern and Western, Turkey	Primiparous mothers (*N* = 60); C‐section; healthy baby; 2 BFH hospitals; 1 hospital with Kangaroo Mother Care (KMC)	Perceived milk supply score higher in KMC than control group; *p* < 0.001	Not available Scale score
RCT (*n* = 8)			
Blixt et al. (2014), Southwest Sweden	Intervention group (IG, *N* = 145): Primiparous women receiving care by midwives and other health professionals Two control groups: CG1 (*N* = 126) and CG2 (*N* = 132)	Among EBF mothers (first 3 mo pp) IG less likely CG1 and CG2 to report SRIM as a BF problem <3 mo pp Among EBF mothers >3 mo pp onward NS difference on SRIM across groups	20% IG <3 mo 56%–60% CG1 and CG2 <3 mo 21% IG ≥ 3 mo 16%–18% CG1 and CG2 ≥3 mo
Chezem et al. (2004), Indianapolis, IN, USA	IG (*n* = 50): home/phone pp BF support by lactation educator CG (*n* = 50): contact information for BF support	Lactation support was protective IG women lower SRIM prevalence than CG (18.5% vs. 47%)	33% Reason for stopping BF
Lewkowitz et al. (2021), Midwest, USA	Low‐income primiparous pregnant mothers (*N* = 87) Smartphone BF app versus control	No association between SRIM and BF app at 2 and 6 wks pp	24.4%‐33.3% BF problem at 2 d pp
Lewkowitz et al. (2018), USA	African American women with overweight/obesity delivering a singleton (*N* = 118). Parenting home intervention with tor without BF between 6 and 12 mo pp	No association between home‐based parenting intervention and SRIM	Not available
Molinero Diaz et al. (2015), Ciudad Real, Spain	Women delivering a healthy baby at a University Hospital (*N* = 100). Intervention; BF nurses' support	Lower SRIM among intervention versus control group (11.4% vs. 26.7%)	19.1% Stopping BF
Pisacane et al. (2005), Naples, Italy	Fathers of healthy newborn (*N* = 280); Intervention group (IG): In‐hospital midwife BF education; Control group (CG): Child care education	Father's BF education At 6 mo pp, less SRIM in IG (12%) than CG (43%), *p* < 0.001	13‐43% BF problem
Ransjo‐Arvidson et al. (1998), Lusaka, Zambia	Mothers with vaginal delivery; healthy newborn, (*N* = 408): IG: midwife visits in their homes 3–42 d pp; CG: visit at 42 d pp	Mother's home BF support Higher SRIM in CG (16%) versus IG (5%)	5‐16% BF problem
Vittoz et al. (2005), Chambery, France	BF mothers; healthy full‐term infant; 3 maternity facilities. CG: usual BF care (*N* = 115); IG: plus paediatrician BF support at 2 wks pp (*N* = 116)	SRIM: 23.4% in IG versus 37.7% in CG	23.4%‐37.7% BF problem
*Retrospective* (*n* = 12)			
Amir and Cwikel (2005), Negev, Israel	205 25–42 years old women with <18 years old children Infant feeding practices telephone survey	Infant loss of interest in BF No association with … SES/Dem	43.9% reason for stopping BF <3 mo
(Hla et al. (2003), Hawaii, USA	Hawaii Vital Records Registry Mailed infant feeding questionnaire (*n* = 667)	SRIM higher among Japanese versus Caucasian women (20.7% vs. 14.2%, *p* < 0.05)	14.2%–20.7% Stopping BF
Kirkland and Fein (2003), National Survey, USA	Infant Feeding Practice Study; BF for at least 1 wk; stopped by 1 year pp (*N* = 758)	Not living in the Midwest and primiparity	32% (41% at 1–2, 23% at 6–12 mo pp) Stopping BF
T. Li et al. (2019), Shanghai, China	BF mothers delivering a healthy full‐term singleton baby at BFH (*N* = 801)	Low maternal self‐efficacy *p* < 0.001	37.1% Perceived insufficient milk
López et al. (2013), Medellín, Colombia	Mothers with infants <24 mo who stopped BF <6 mo (*N* = 303)	Less SRIM among 15–18 years old (26.32%) versus <14 years old (40%), versus 19–30 years old (39.9%) versus >30 years old (38.5%) mothers, *p* = 0.065 SRIM lowest for 4–5 mo (8.3%) versus at birth (34.9%), 1 mo (35.1%), 2 mo (39.2%), 3–4 mo (35.1%) infants, *p* = 0.093	45.9% Stopping BF
Mallan et al. (2018), Brisbane, Australia	Women recruited prenatally (*n* = 715); retrospective interview at 4 mo pp	Higher SRIM among overweight (32%) compared with nonoverweight (23%) women, *p* = 0.10	25% BF problem 49% introducing formula
Moll Pons et al. (2012), Palma de Mallorca, Spain	Mothers with infants 15, 30, 90, 180 d old (*N* = 72); telephone survey	SRIM higher at 1 (75.6%) versus 3–6 (18.2%) mo pp. SRIM cues: baby crying, baby hungry, baby's slow weight gain	18.2%–75.6% Partial BF
Negayama et al. (2012), Japan, France, USA	Mothers with 4–20 mo old infants in Japan (*N* = 310), France (*N* = 756), US (*N* = 222)	Country More SRIM among Japanese versus French and US women (*p* = 0.001)	SRIM Stop BF across 1st year Japan: 16.2%–50.6% France: 10.6%–23.5% US: 20%–30.4%
Sahin et al. (2013), Kayseri Province, Turkey	Mothers of 24–60 mo old children attending family health centres (*N* = 500)	SRIM RF: housewife; poverty; C‐section; starting BF > 1 h pp; BF daily frequency <8	34.2% BF problem
Tracz and Gajewska (2020), 2018 National survey, Poland	BF mothers with 6–18 mo old infants (*N* = 1024)	SRIM by infant age: <1 mo—25.3%, 1–2 mo—29.6%, 3–5 mo—32.6%, 6–8 mo—11.7%, ≥9 mo—0.9%, *p* < 0.001	41% Stopping EBF
Whipps and Demirci (2021) National survey, USA	6‐year FUP IFPS II mothers (*N* = 1460); 1 (*N* = 350) or 2 (*N* = 78) subsequent children	SRIM by child: index child, 66.6%; subsequent child 1; 65.7%, subsequent child 2; 64.1%	66.6% Stopping BF
Williams et al. (1999), Vancouver, Canada	Mothers of full‐term, healthy infants participating in an iron deficiency study (*N* = 434)	28% of Caucasian reported SRIM as a reason for using formula versus 4% of non‐Caucasian, *p* < 0.0001 SRIM by age: <3 mo, 10%; 3–6 mo, 29%; >6 mo, 4%, *p* < 0.0001	4%–29% Stopping BF

Abbreviations: BF, breastfeeding; CI, confidence interval; d, days; EBF, exclusive breastfeeding; FUP, follow‐up; IFPS, infant feeding practices study; mo, month; OR, odds ratio; pp, postpartum; SRIM, self‐reported insufficient milk; wks, weeks.

**Table 4:** Delayed onset of lactation (DOL) study characteristics by study design

Author (year), country	Population/setting	Key findings	DOL (%)
*Cross‐sectional* (*n* = 6)			
Brownell et al. (2012), National Survey, USA	BF mothers with healthy singleton infant Infant Feeding Practices Survey II survey at 3 wks pp (*N* = 2491)	Poverty; South Region; no BF experience; Not BF on demand; obstetrician/paediatrician unsupportive of BF; not having a low Intervention vaginal birth; epidural anaesthesia; Not rooming in all the time; Receiving in‐hospital BF support; Returned to work >6 mo	23.2%
Chertok and Sherby (2016) Israel	BF self‐efficacy assessed Mothers with and without GDM, full‐term infant (*N* = 67)	Gestational Diabetes Mellitus 31.3% GDM versus 8.6% without GDM reported DOL, *p* = 0.029	8.6%–31.3%
Haile et al. (2017), National Survey, USA	Women with healthy term infants (*N* = 2053) Infant Feeding Practices Survey II (2005–2007)	Non‐Hispanic White women with high GWG; poverty; C‐section; pain medication/anesthesia at delivery	24% By GWG: inadequate 22.4% adequate 19.8% excessive 26.7%
Hruschka et al. (2003), Four communities in Guatemala	Mothers of infants born between 1996 and 1999 in four communities (*N* = 328)	Formula supplementation	10.1% By community: A 7.8%, B 9.6%, C 15.4%, D 10%
Kung and Bajorek (2008), Sydney, Australia	Mothers delivering full‐term, singleton infant (*N* = 75)	Prolonged stage II labour	39%
Scott et al. (2007), Perth, Australia	Women participating in an Infant Feeding Study (PIFS II) who delivered at two public hospitals between mid‐Sept. 2002 and mid‐July 2003. Participants completed a self‐administered survey at the hospital or right after discharge (*n* = 453)	DOL Rf's: C‐section OR: 2.40; 95% CI (1.28–4.51) *p* = 0.007 and primiparity 3.16 (1.58–6.33) *p* = 0.001. There was a not significant tend among overwt./obese women to report DOL (40.8%) versus nonoverwt/obese (32.1%)	11.7% (53/453)
*Prospective* (*n* = 12)			
Casey et al. (2019), Indiana, USA	Primiparous pregnant women at 22 wks (*N* = 50) and 32 wks of gestation (*N* = 44) Maternal sleep: wrist actigraphy	Suboptimal maternal sleep Women with better maternal sleep and more stable night sleep time during pregnancy, less likely to report DOL	31%
Chapman and Perez‐Escamilla (2000), Hartford, CT, USA	BF mothers with c‐section delivery of a healthy infant (*N* = 57)	BF initiation >105 min; unscheduled c‐section; breast pumping before OL (among primiparae)	35%–40%
Dewey et al. (2003), Davis, CA, USA	Women with full‐term infant (*N* = 280) Assessments conducted at hospital, 3, 5, 7, and 14 pp	Long stage II labour; C‐section; high BMI; flat/inverted nipples; excessive perinatal weight loss	22%
Flaherman et al. (2016), Hershey, PA, USA	BF mothers with infants born ≥34 wks (*N* = 1107), assessed: hospital, 2 wks pp	Excessive perinatal weight loss (EWL), maternal anxiety	Not reported
Huang et al. (2019), Wuhan, China	BF women who delivered a full‐term newborn (*N* = 3282) Subsample from the TMCHC study of pregnant women	Significant relationship with GWG quartile	18.4%
Huang et al. (2020), Wuhan, China	BF women who delivered a full‐term newborn (*N* = 2877) Subsample from the TMCHC study of pregnant women	Low LATCH scores; formula during first 72h; primiparous; GDM; higher GWG; lower gestational age at delivery; higher birth weight; higher length at birth	17.9%
Matias et al. (2010), Canto Grande, Peru	Primiparous women giving birth at a BFH (*N* = 171); followed first 4 d pp	Low Apgar Score	17%
Mohebati et al. (2021), Mexico City, Mexico	Primiparous mothers; not working (*N* = 475); full‐term; hospital, 1–4 wks pp	Lower maternal responsiveness to crying; lower BF frequency at 1 wk pp; C‐section delivery	20%–41% vaginal versus C‐section delivery
Nommsen‐Rivers et al. (2010), Davis, CA, USA	Pregnant primiparae, single term newborn, initiated BF (*N* = 431); assessed: at 0, 3, 7 d pp, phone follow‐up	≥30 y old, maternal overweight or obesity, BW > 3600 g, lack of nipple discomfort 0–3 d pp, BF ≤2 times in first 24 h	44.3%
Preusting et al. (2017), Tampa Florida, USA	English speaking pregnant women planning BF, BFH (*N* = 216)	Higher maternal age, pre‐pregnancy BMI, gestational weight gain, epidural anesthesia	46.4%–57.9% BMI < 30 versus ≥ 30 kg/m^2^
Rocha et al. (2020), Southeastern, Brazil	Primiparous mothers, healthy term infant, planning EBF (*N* = 224), BFH	Alcohol during pregnancy; maternal age; in‐hospital formula; post‐natal depression	18.8%
Sellen et al. (2004), Rural communities in Guatemala	Women were interviewed at home, 8 d pp, 1 and 3 mo pp; cohort born between June 1996 and July 1998	DOL Rfs: Maternal obesity (BMI > 30) 2.7 (1.1–6.5) Maternal age (>median, 26 years) 1.8 (1.04–3.0) Short preceding birth «2 years) 2.1 (1.2–3.7)	Not available
*Quasi‐experimental* (*n* = 1)			
Nommsen‐Rivers et al. (2009), Davis, CA, USA	Primiparous low‐income women, able to BF, full‐term healthy baby DC (*n* = 44) SC (*n* = 97); assessed at hospital, and at 3 d, 6 wks pp via phone	DC more likely (vs. SC) to have timely OL (58.3% vs. 45.2%) and no milk supply concerns at 3 d pp 71.8% versus 62.4%). DOL RF's: milk supply concerns; higher maternal BMI; SC group, not avoiding pacifier; >2 times formula during first 48 h pp	41.7%–54.8%
RCT (*n* = 2)			
Fok et al. (2019), Singapur	Women with full term deliveries IG: breast milk expression within 1 h pp followed by regular expression with direct BF (*n* = 31) CG: Direct BF without regular pump expression (*n* = 29)	Lack of breast milk expression CG more likely to have DOL than IG (*p* < 0.05).	42%–69%
Turok et al. (2017), New Mexico and Utah, USA	Participants planning to use a levonorgestrel intrauterine device as contraceptive. method post‐partum. Women assigned to the immediate (*N* = 125) or delayed (*N* = 103) intrauterine device group	DOL: 9% in immediate versus 6% in delayed IUD group, *p* = 0.46	9%–6% DOL > 120 h p
*Retrospective* (*n* = 1)			
Mallan et al. (2018), Brisbane, Australia	Women recruited antenatally (*n* = 715); interviewed at 4 mo about their infant feeding practices during 1st mo pp	DOL NS between overweight (25%) and nonoverweight (20%) women. DOL as reason for formula at 1 mo pp: overweight (55%) versus nonoverweight (48%)	21%

Abbreviations: BF, breastfeeding; BG, bottle group; BMI, body mass index; CG, control group; d, days; DC, doula care; DOL, delayed onset of lactation; EBF, exclusive breastfeeding; GDM, gestational diabetes mellitus; GWG, gestational weight gain; IG, intervention group; pp, postpartum; SC, Standard of care; TMCHC, Tongji Maternal and Child Health Cohort.

### 3.3 Quality of evidence

#### 3.3.1 Cross‐sectional and retrospective studies

**Figure 2:**
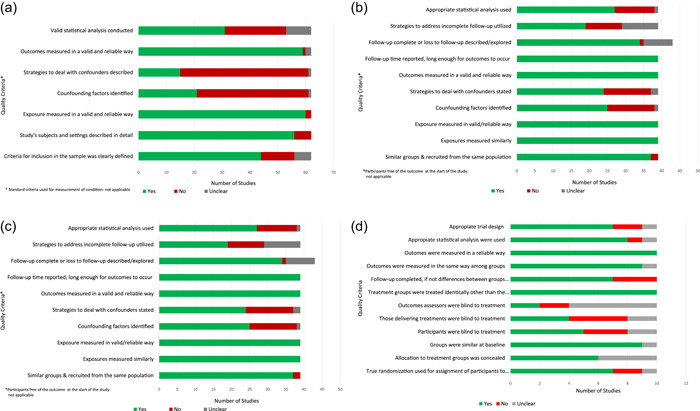
(a) Study quality analysis: Cross‐sectional and retrospective studies (*n* = 62). (b) Study quality analysis: Prospective studies (*n* = 39). (c) Study quality analysis: Quasi‐experimental studies (*n* = 19). (d) Study quality analysis: Randomised controlled trials (*n* = 10)

The quality of cross‐sectional and retrospective studies (*n* = 62) was based on seven items (Figure 2a). Most studies described the setting well and measured the exposure and outcome in valid and reliable ways (three items). However, half of the studies had deficiencies with statistical analyses mainly due to the lack of multivariate statistical approaches and hence the inability to properly account for confounding.

#### 3.3.2 Prospective studies

Of the 11 items included in the assessment of the prospective studies (*n* = 39), most studies (37 out of 39) met five of the criteria. Twelve studies were classified as not having performed adequate statistical analyses, and 20 did not address missing data due to incomplete follow‐up (Figure 2b).

#### 3.3.3 Quasi‐experimental studies

Among the nine items that were used to assess the quality of the nine quasi‐experimental studies, a significant proportion had deficiencies in statistical analyses mainly due to not properly accounting for potential confounding (Figure 2c).

#### 3.3.4 Experimental studies

Five of the 10 RCTs met the criteria for at least 9 items out of 12 items. However only two met the outcomes assessors; four the intervention; five met the participant allocation blindness criteria; seven met the trial design, randomisation, and follow‐up criteria, and eight met the statistical analysis criteria (Figure 2d).

### 3.4 Risk factors for SRIM and DOL

#### 3.4.1 SRIM

There were 63 risk factors identified and categorised into seven domains: socioeconomic and cultural; demographic; psychosocial and behavioural; health care systems; biomedical; breastfeeding knowledge, styles, and problems, and maternal lifestyles (Supporting Information Appendix SA). The following section summarises individual or groups of risk factors that were found to be consistently associated with SRIM, meaning that over half of studies examining a specific risk factor documented the significant association (Tables 3 and 5).

**Table 5:** Risk factors consistently identified for self‐reported insufficient milk and delayed onset of lactation. Systematic review

	SRIM	DOL
Risk factors	***n*/*N***	***n*/*N***
Socioeconomic and demographic		
Low maternal education	5 out of 6	
Household poverty	4 out of 5	
Maternal employment	3 out of 3	
Maternal age (Younger for SRIM and older for DOL)	5 out of 6	3 out of 3
Primiparity	5 out of 9	5 out of 5
Social support, Psychosocial and Behavioural		
Lack of family support	4 out of 4	
Infant crying/fussiness/baby behaviour	15 out of15	
Perceived poor sucking	3 out of 3	
Low maternal BF self‐efficacy	10 out of 10	
Maternity ward practices and BF counselling		
Delayed BF initiation	3 out of 4	
In‐hospital CMF introduction	4 out of 4	4 out of 4
Other practices inconsistent with Ten Steps	5 out of 5	3 out of 3
Lack of breastfeeding counseling	6 out of 7	
Biomedical		
C‐section	5 out of 6	3 out of 3
Epidural anesthesia		3 out of 3
Prolonged stage II labour		3 out of 3
Maternal overweight/obesity	6 out of 6	4 out of 4
Excessive gestational weight gain		4 out of 4
Excessive newborn weight loss/perceived poor growth	5 out of 5	
Low birth weight or prematurity	3 out of 4	
Poor maternal physical and mental health	7 out of 7	
Breastfeeding challenges		
Early breastfeeding problems	5 out of 6	
Delayed onset of lactation	4 out of 4	NA
Early introduction of solids and/or CMF	4 out of 4	
Maternal lifestyles		
Anxiety, depression, poor sleep, alcohol or tobacco use		4 out of 4

Abbreviations: DOL, delayed onset of lactation; *n*, number of studies finding association; *N*, total number of studies; NA, not applicable; SRIM, self‐reported insufficient milk.

##### 3.4.1.1 Socioeconomic and demographic

The SRIM risk factors very consistently identified were low maternal education (five of six studies examining this risk factor) (Gokceoglu & Kucukoglu, 2017; Menekseet al., 2021; Robert et al., 2014; Rodrigo et al., 2019; Segura‐Millán et al., 1994), primiparity (five of nine studies) (Brownell et al., 2012; Hillervik‐Lindquist, 1992; Keemer, 2013; Kent et al., 2021; Kirkland & Fein, 2003), maternal employment (three of three studies) (Chuang et al., 2007; Sahin et al., 2013; Sun‐Hee, 2019), and younger maternal age (five of six studies) (Amine et al., 1989; Brown et al., 2014; Gokceoglu & Kucukoglu, 2017; López et al., 2013; Rodrigo et al., 2019). On the other hand, having a high household income was found to be a protective factor for SRIM in four out of five studies (Gokceoglu & Kucukoglu, 2017; Herrera, 2008; Li et al., 2008; Sahin et al., 2013). All studies (*n* = 8) comparing ethnic/racial groups within‐countries or mothers across countries, and three of four studies comparing area of residence characteristics including urban/rural, found significant associations of these demographic variables with SRIM tending to be comparatively higher among women of relatively disadvantaged groups, at higher SRIM risk were: Jewish versus Arab women (Heldenberg et al., 1993); Japanese versus Caucasian women (Hla et al., 2003); Hispanic (vs. African American, White) women (Hurley et al., 2008; Rozga et al., 2015); Hispanic (vs. White) women (Li et al., 2008); non‐Caucasian (vs. Caucasian) women (Kent et al., 2021; Williams et al., 1999); rural versus urban (Mosha et al., 1998), Japanese versus French/US (Negayama et al., 2012), outside United States Midwest versus other parts of the United States (Kirkland & Fein, 2003). Findings from 19 studies did not show clear patterns of associations between SRIM and infant age (Table 3).

##### 3.4.1.2 Social support, psychosocial and behavioural

SRIM protective factors related to social support and psychosocial indicators were identified.

All four studies examining family (Chuang et al., 2007; Hill & Aldag, 1991; Rodrigo et al., 2019) or father (Hill & Aldag, 1991) support found these factors to be protective against SRIM. Maternal BF self‐efficacy was protective against SRIM in all studies examining it (*n* = 10) (Galipeau et al., 2017; Gokceoglu & Kucukoglu, 2017; Gumussoy et al., 2020; Hill & Aldag, 1991; T. Li, Guo et al., 2019; McCarter‐Spaulding & Kearney, 2001; Menekse et al., 2021; Otsuka et al., 2008; Sandhi et al., 2020; Segura‐Millán et al., 1994).

Infant behaviours were consistently associated with SRIM across many studies. Specifically, all studies addressing infant irritability, fussiness, crying (15 out of 15 studies) (Galipeau et al., 2017; Graffy, 1992; Hill & Aldag, 1991; McCann & Bender, 2006; Mohebati et al., 2021; Moll Pons et al., 2012; Monteiro et al., 2011; Perez‐Escamilla et al., 1992; Segura‐Millán et al., 1994; Tully & Dewey, 1985; Wood et al., 2017), baby's loss of interest in breastfeeding or breast refusal (Amir & Cwikel, 2005; Hill & Aldag, 1991; O'Sullivan et al., 2015), baby not satisfied with feeding (Cooke et al., 2003; Huang, Lee, et al., 2009; Moll Pons et al., 2012; Monteiro et al., 2011), or perceived poor sucking (three out of three studies) (Galipeau et al., 2017; Hill & Aldag, 1991; Huang, Lee, et al., 2009; Mizuno et al., 2004) identified them as SRIM risk factors (Huang, Gau, et al., 2009).

##### 3.4.1.3 Maternity ward practices and BF counselling

Delivering in baby‐friendly hospitals or maternity practices aligned with the WHO/UNICEF Ten Steps to Successful Breastfeeding (Pérez‐Escamilla et al., 2016) were consistently identified as protective against SRIM (Table 3). Ten steps practices found to be protective against SRIM included timely BF initiation (three out of four studies) (Lin et al., 2011; Menekse et al., 2021; Sahin et al., 2013), no in‐hospital CMF supplementation (four out of four studies) (Huang, Gau, et al., 2009; Lin et al., 2011; Menekse et al., 2021; Tully & Dewey, 1985), and BF counselling (six out of seven studies) for mothers (Blixt et al., 2014; Chezem et al., 2004; Molinero Diaz et al., 2015; Nommsen‐Rivers et al., 2009; Silbert‐Flagg et al., 2020; Vittoz et al., 2005) and fathers (Pisacane et al., 2005). Five additional 10 steps‐related practices were found to be protective against SRIM: pre‐natal BF support (Ume et al., 2014), skin‐to‐skin contact (Sandhi et al., 2020), rooming‐in (Perez‐Escamilla et al., 1992), Kangaroo care (Yilmaz et al., 2020) and being born in a Baby‐Friendly Hospital (Robert et al., 2014).

##### 3.4.1.4 Biomedical

Cesarean delivery (C‐section) and maternal overweight were identified as SRIM risk factors in five out of six studies (Donmez & Korgali, 2021; Kent et al., 2021; Rodrigo et al., 2019; Sahin et al., 2013; Sun‐Hee, 2019) and six out of six studies, respectively (Jarlenski et al., 2014; Kair & Colaizy, 2016; Mallan et al., 2018; Mok et al., 2008; O'Sullivan et al., 2015).

All seven studies addressing poor mental (Flaherman et al., 2016; Hazrat et al., 2017; Hillervik‐Lindquist, 1992; Rodrigo et al., 2019) or physical health (Henly et al., 1995; Hill & Aldag, 1991; Rodrigo et al., 2019) (*n* = 5) identified poor maternal well‐being as a SRIM risk factor.

Excessive newborn weight loss or perception that infant was not growing well were also identified as SRIM risk factors (five out of five studies) (Flaherman et al., 2016; Hill & Aldag, 1991; Hillerviklindquist et al., 1991; Kent et al., 2021; Moll Pons et al., 2012; O'Sullivan et al., 2015). Three of four studies identified low‐birth‐weight or pre‐maturity as SRIM risk factors.

##### 3.4.1.5 Breastfeeding challenges

DOL was a risk factor for SRIM in all studies examining it (*N* = 4) (Kent et al., 2021; Mohebati et al., 2021; O'Sullivan et al., 2015; Segura‐Millán et al., 1994). Early BF difficulties including sore nipples were also risk factors for SRIM (five out of six studies) (Mohebati et al., 2021; O'Sullivan et al., 2015; Segura‐Millán et al., 1994; Whichelow, 1979).

Additional risk factors for SRIM included early introduction of CMF (Segura‐Millán et al., 1994), displacement of breast milk by solids between BF episodes (Hillerviklindquist et al., 1991) or mixed feeding (Huang, Lee, et al., 2009; Kent et al., 2021) (four out of four studies).

**Figure 3:**
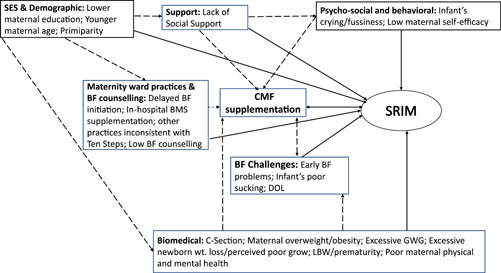
Self‐reported insufficient milk (SRIM) conceptual framework. SRIM is determined by distal (socioeconomic and demographic), intermediate (social support, psychoemotional and baby behaviours, maternity ward practices, biomedical), and proximal (commercial milk formula [CMF] supplementation, and BF challenges) factors. Dotted lines indicate relationships not tested in the systematic review. DOL, delayed onset of lactation; SES, socioeconomics status

In sum, a graphic synthesis of our findings suggests that SRIM is determined by distal (socioeconomic and demographic), intermediate (social support, psychoemotional and baby behaviours, maternity ward practices, biomedical) and proximal (CMF supplementation, and BF challenges) factors (see conceptual framework in Figure 3).

#### 3.4.2 DOL

There were 31 risk factors for DOL classified into seven domains; socioeconomic, demographic, psychosocial and behavioural; health care systems; biomedical, breastfeeding knowledge, styles and problems; and maternal lifestyles (Supporting Information Appendix SB). The following section summarises findings for risk factors for which there were at least three studies addressing them (Tables 4 and 5).

##### 3.4.2.1 Socioeconomic and demographic

Studies identified household poverty (Brownell et al., 2012; Haile et al., 2017) maternal employment (Brownell et al., 2012) were socioeconomic indicators associated with DOL in our review. Demographic factors such as primiparity was identified as a risk factor in three studies (Brownell et al., 2012; Huang, Li et al., 2020; Scott et al., 2007), and in two more were linked to an interaction, only among primiparous who pumped (Chapman & Perez‐Escamilla, 2000) or among primiparas who delivered a large infant (Dewey et al., 2003). Older maternal age (Nommsen‐Rivers et al., 2010; Preusting et al., 2017; Rocha et al., 2020) as DOL risk factors.

##### 3.4.2.2 Maternity ward practices and BF counselling

Early introduction of CMF was identified as a risk factor for DOL in all four studies examining it (Hruschka et al., 2003; Huang et al., 2020; Nommsen‐Rivers et al., 2009; Rocha et al., 2020). Three studies identified rooming‐in (Brownell et al., 2012), timely initiation of BF (Chapman & Perez‐Escamilla, 2000), and BF counselling (Brownell et al., 2012) as protective against DOL.

##### 3.4.2.3 Biomedical

Three studies identified C‐section (Chapman & Perez‐Escamilla, 2000; Dewey et al., 2003; Haile et al., 2017) as a risk factor for DOL. Four studies identified maternal overweight or obesity as a DOL risk factor (Dewey et al., 2003; Nommsen‐Rivers et al., 2010; Preusting et al., 2017; Sellen et al., 2004). Likewise, all four studies examining it consistently identified excessive gestational weight gain as a risk factor for DOL (Haile et al., 2017; Huang et al., 2019, 2020; Preusting et al., 2017).

All six studies focusing on labour duration found that prolonged Stage II labour (Chapman & Pérez‐Escamilla, 1999b; Dewey et al., 2003; Kung & Bajorek, 2008) and epidural anesthesia (Brownell et al., 2012; Haile et al., 2017; Preusting et al., 2017) identified these obstetric outcomes as risk factors for DOL.Two studies found that excessive newborn weight loss was associated with DOL (Dewey et al., 2003; Flaherman et al., 2016).

##### 3.4.2.4 Maternal health and lifestyles

Two studies found that maternal anxiety and depression were risk factors for DOL (Flaherman et al., 2016; Rocha et al., 2020). Studies also showed that poor maternal sleep (Casey et al., 2019), alcohol (Brownell et al., 2012) and tobacco (Brownell et al., 2012) use were risk factors for DOL.

**Figure 4:**
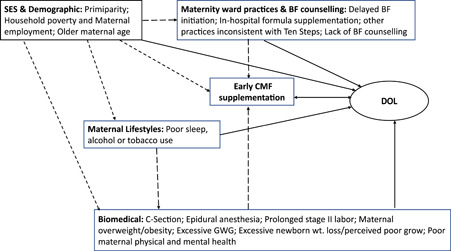
Delayed onset of lactation (DOL) conceptual framework. DOL is determined by distal (socioeconomic and demographic), intermediate (social support, maternal lifestyles, maternity ward practices and breastfeeding counselling, biomedical) and proximal (commercial milk formula (CMF) supplementation) factors. Dotted lines indicate relationships not tested in systematic review. SES, socioeconomics status

In sum, a graphic synthesis of our findings suggests that DOL is determined by distal (socioeconomic and demographic), intermediate (social support, maternal lifestyles, maternity ward practices, and breastfeeding counseling, biomedical) and proximal (CMF) supplementation, and factors (see conceptual framework in Figure 4).

## 4 DISCUSSION

Our highly comprehensive systematic SRIM review, the first of its kind as far as we know, indicates that DOL and SRIM continue to be highly prevalent and that both SRIM and DOL are associated with multiple factors distributed across socioeconomic, demographic, support systems, health care systems, psychosocial and behavioural, and breastfeeding and human lactation domains.

Our findings showed that delayed BF initiation, separation rather than rooming‐in, in‐hospital CMF supplementation, lack of BF counseling, and other maternity practices inconsistent with the Ten Steps were risk factors for both DOL and SRIM. Since not following these practices was associated with an increase in the risk for DOL and SRIM, we strongly recommend that future studies examining the impact of the Baby‐Friendly Hospital Initiative Ten Steps also include DOL and SRIM as primary outcomes.

Our review strongly affirmed the multifactorial nature of SRIM and the importance of designing interventions to address the constellation of risk factors identified. We documented a relationship of maternal breastfeeding self‐efficacy with a reduced risk of SRIM and longer breastfeeding duration. Hence, it is important for breastfeeding counseling programmes to prepare women from pregnancy and the early post‐partum period by building their confidence toward establishing an ample milk supply to nourish their infants. Counseling may be especially important for primiparous women, as they were consistently found to be at higher risk of SRIM.

Our review strongly suggests that to prevent SRIM it is important to improve caregivers' and healthcare professionals' understanding and management of baby behaviours perceived to be ‘difficult’ through responsive feeding approaches (Pérez‐Escamilla et al., 2021), such as fussiness/crying, since such perceptions can lead to unnecessary CMF supplementation (Vilar‐Compte et al., In Press) very soon after birth which, in turn, is a major risk factor for the pre‐mature termination of EBF and any BF (Pérez‐Escamilla et al., 2019).

Our findings show that SRIM during the very early post‐partum period can be driven by DOL or not having received adequate information on the expected limited milk production during the colostrum stage of lactation. Given that maternal stress is a risk factor for DOL, it is important to provide strong psychoemotional support to women as a routine part of pre‐natal, perinatal and post‐natal breastfeeding counseling. Our review also suggests that psychoemotional support from family members, including fathers, is needed to prevent SRIM. It is likely that psychoemotional support from counsellors, family, friends, and healthcare providers as part of breastfeeding programmes will lead to improving maternal BF self‐efficacy, which was consistently found in our review to be a protective factor against SRIM.

Regarding biomedical factors including obstetric practices, our review consistently showed that maternal pre‐pregnancy obesity was consistently identified as a risk factor of DOL. Furthermore, C‐sections and maternal overweight or obesity were risk factors for both DOL and SRIM. Therefore, interventions are needed to both prevent unnecessary C‐sections and maternal excessive body fat and weight gain during pregnancy and to provide additional needed lactation support to women exposed to these highly prevalent risk factors (Pérez‐Escamilla et al., 2019).

Consistent with a previous review (Rollins et al., 2016) we found that women of lower socioeconomic status, as reflected by household income and education level, are more likely to report DOL and SRIM. Likewise, there were differences in risk between ethnic/racial groups or urban versus rural areas within countries and when comparing women across countries. Hence, addressing this global public health concern should be done through an equity lens focusing on the social determinants of health (Pérez‐Escamilla, 2020; Pérez‐Escamilla & Sellen, 2015) mediating the relationship between poverty and SRIM.

Overall, our review supports that SRIM may be a concern that starts since the colostrum stage when there is very little milk production, leading to the unnecessary introduction of CMF during the early neonatal period (Pérez‐Escamilla et al., 2019). This and the reduction of nursing frequency may lead to an actual breastmilk insufficiency (Karall et al., 2015; Pérez‐Escamilla et al., 2019) and the pre‐mature termination of EBF and any BF (Pérez‐Escamilla et al., in Press). Our findings that baby behaviours conveying infant distress are risk factors for SRIM are consistent with a recent systematic review documenting the relationship between baby behaviours and infant feeding decisions by their caregivers (Vilar‐Compte et al., in Press).

Our review has several limitations. First, the study quality assessment showed that statistical analyses for SRIM studies were often inadequate mainly because multivariable analyses were not conducted to adjust for potential confounders, calling for the need for major improvements in research quality in this area moving forward. Second, given that only one study did not find any risk factors for SRIM (Ducket et al., 1998), it is possible that publication bias was present. However, this is unlikely as there were very few studies for which SRIM was the primary outcome. In most studies from which SRIM risk factors were identified, the primary outcomes were BF or EBF prevalence or duration. Third, we could not quantify the relative importance of SRIM risk factors due to the small number of studies per risk factor, the lack of studies designed to answer these questions, and the strong heterogeneity in study designs, outcomes definition, and contexts examined. Hence, the original decision made to write a systematic review protocol without meta‐analysis was well‐founded. Fourth, we could not present findings by the level of economic development of countries or rural versus urban residence given the limited number of studies per risk factor.

In the previous literature, PIM was used to refer to ‘perceived insufficient milk’. We chose not to use this term in our review because ‘perception’ has been taken to imply that the milk insufficiency mothers are reporting is often times not real and simply given as a socially acceptable excuse by women. Instead, we coined the term SRIM to describe more accurately the phenomena of interest in this article and it is non‐judgmental.

As stated above, it should not be assumed that SRIM does not reflect real milk insufficiency (Pérez‐Escamilla et al., 2019; Stuebe, 2021). It is important for the hydration status of all newborns to be closely monitored. It is also important to empower women to work together with their health care providers to ensure that their infants are growing and developing well. It is crucial for all women and their infant feeding support networks to receive anticipatory guidance and counseling starting at the beginning of pregnancy and continuing through the perinatally and post‐natally periods on the different stages of lactation (lactation secretory activation, establishment, and maintenance; Boss et al., 2018) and what to expect with regard to milk production during each of them. Specifically, counsellors should prepare mothers what to expect with regard to their milk production before, during, and after the onset of lactation; allay fears about not having enough milk; counsel them on how to establish if their infants are getting enough nourishment through breastfeeding; and support them in learning and properly applying breastfeeding techniques that are key to promoting sufficient milk supply and managing SRIM and DOL as necessary—for example, through increased nursing frequency during infant growth spurts (Galipeau et al., 2018). All considered, this is the only way caregivers can be reassured that the breastfed infant is receiving adequate nourishment (Pérez‐Escamilla et al., 2019).

### 4.1 Data gaps and multidisciplinary research recommendations

We identified the following key gaps in our knowledge of SRIM and DOL and research priorities.

First, there is a strong need for studies in low‐ and low‐middle‐income countries. These studies should focus on the potential role of maternal malnutrition, both under and overweight and micronutrient deficiencies, on DOL and SRIM, and use objective measures of breast milk volume and quality. It is particularly important to prioritise SRIM and DOL prevention among infants less than 6 months old to prevent wasting taking into account available BF assessment tools for at‐risk and malnourished infants aged under 6 months old (Brugaletta et al., 2020; UNICEF, 2021).

Second, studies are needed to examine the longer‐term implications of SRIM on the risk of illness episodes; in addition to its potential impact on the anthropometric status and growth trajectories of infants.

Third, there is a need to design experimental studies across different sociocultural contexts and maternity care settings that have as primary aim understanding how to prevent SRIM. Interventions based on the Ten Steps of the Baby‐Friendly Hospital Initiative and breastfeeding counseling are promising.

Fourth, SRIM studies need to help understand how to prevent SRIM by addressing concerns about baby behaviour, improving maternal BF self‐efficacy, and differentiating perceptions related to quantity versus quality of breast milk. This information is crucial to properly design effective BF counseling interventions across different contexts and settings.

Fifth, given that up to 5% of women may have basic physiological difficulties with breastfeeding (Pérez‐Escamilla et al., 2019) and that additional women may experience subclinical and clinical lactation challenges such as subclinical mastitis or plugged ducts or difficulties related to overweight or obesity, it is important to develop clinical screening tools to detect those women before they deliver their infants so that their breastfeeding needs can be supported, including by alternative infant feeding options (Stuebe, 2021).

Sixth, it is essential to better understand how the CMF industry exploits fears that women have about DOL and SRIM as part of their marketing strategies, which end up undermining women's BF self‐efficacy (Piwoz & Huffman, 2015; World Health Organization & UNICEF, 2022).

Seventh, understanding must be improved on how best to support the onset of milk production and other breastfeeding needs of women who are overweight or obese (Chapman et al., 2013, 2016; Garner et al., 2017; Martinez et al., 2016).

Eighth, it is key to reach a consensus on the best ways to measure SRIM to track trends in prevalence and risk factors through monitoring and surveillance systems.

### 4.2 Practice implications

Our review has several important public health practice implications. First, pre‐natal, perinatal, and post‐natal health care professionals and community BF counsellors need better training on risk factors for SRIM and DOL, and how to support women's establishment and maintenance of milk supply (Pérez‐Escamilla et al., 2019). Second, maternity care systems need to be empowered to facilitate timely breastfeeding initiation and prevention of pre‐lacteal feeding, not only to prevent DOL and SRIM but also to improve BF outcomes and infant survival (Perez‐Escamilla et al., In Press). Health care providers engaged in maternity services should receive better training on building BF self‐efficacy in women by improving their ability to understand and manage baby behaviours perceived as ‘difficult’ such as fussiness/crying and intermittent sleep (Vilar‐Compte et al., In Press) through responsive feeding approaches (Pérez‐Escamilla et al., 2021). Although we still lack understanding about how to prevent or control these risk factors across different contexts, it is clear that future interventions to prevent DOL and SRIM need to prepare for infant feeding during pregnancy and rectify infant feeding practices in maternity service systems consistent with the Baby‐Friendly Hospital Initiative Ten Steps (Pérez‐Escamilla et al., 2016).

## CONFLICTS OF INTEREST

The authors declare no conflicts of interest.

## AUTHOR CONTRIBUTIONS

Sofia Segura‐Pérez and Rafael Pérez‐Escamilla led the conceptualising and drafting of the protocol for the systematic review, reviewed abstracts, titles and manuscripts, extracted study data, assessed study quality and drafted the full manuscript. Misikir Adnew contributed with titles screening and full‐text reviews. Rafael Pérez‐Escamilla provided guidance in decisions to include or exclude specific studies when consensus was not initially reached between Sofia Segura‐Pérez and Misikir Adnew. Kate Nyhan developed and tested the search strategy, conducted the search, and contributed to defining quality assessment tools. Linda Richter, Elizabeth C. Rhodes, Amber Hromi‐Fiedler, Mireya Vilar‐Compte, and Misikir Adnew contributed to the conceptualisation and draft of the protocol for the systematic review, supported manuscript development, and provided a critical review of the full manuscript. All authors read and approved the submitted version of the manuscript.

## Supporting information

Supporting Information.

Supporting Information.

## Data Availability

The data that support the findings of this study are available from the corresponding author upon reasonable request.
