# Supplementary material for: Risk factors for self‐reported insufficient milk during the first 6 months of life: A systematic review
Source: Matern Child Nutr. 2022 Mar 28;18(Suppl 3):e13353. doi: 10.1111/mcn.13353 (PMC9113468; doi:10.1111/mcn.13353)
Supplement: Supplementary file 1 — Supporting Information. [file MCN-18-e13353-s002.docx]

**Online Appendix A- SRIM Risk Factors**

| Table. Summary of factors associated with self-reported insufficient milk (SRIM) | | | | |
| --- | --- | --- | --- | --- |
| Domain | SRIM Risk Factors | # of studies | | |
|  |  | Risk | Protection | Neutral |
| **Socio-economic and cultural** |  |  |  |  |
| 1. | Higher maternal education | 0 | 5  (Gokceoglu & Kucukoglu, 2017; Menekse, Tiryaki, Karakaya Suzan, & Cinar, 2021; Robert, Coppieters, Swennen, & Dramaix, 2014; Rodrigo, Rodrigo, Liyanage, Hatahagoda, & Hewavitharana, 2019; Segura-Millán, Dewey, & Perez-Escamilla, 1994) | 1  (Duckett et al., 1998) |
| 2. | Higher paternal education | 0 | 1  (Robert et al., 2014) | 0 |
| 3. | Higher household income | 1  (Brown, Dodds, Legge, Bryanton, & Semenic, 2014) | 4  (Gokceoglu & Kucukoglu, 2017)  (Middle-income (vs. lower or higher) (Herrera, 2008; R. Li, Fein, Chen, & Grummer-Strawn, 2008; Sahin et al., 2013) | 0 |
| 4. | Maternal employment | 3  Taiwan born (Chuang et al., 2007; Sahin et al., 2013; Sun-Hee, 2019) | 1  Born outside Taiwan (Chuang et al., 2007) | 0 |
| 5. | Religion | 1  Muslim (vs. non-Muslim) (Einterz & Bates, 1994) | 0 | 0 |
|  |  |  |  |  |
| **Demographic** |  |  |  |  |
| 6. | Primiparity | 5  (Brownell, Howard, Dozier, & Lawrence, 2012; Hillervik-Lindquist, 1992; Keemer, 2013; Kent et al., 2021; Kirkland & Fein, 2003) | 3  (Forman et al., 1992; Segura-Millán et al., 1994; Tully & Dewey, 1985) | 1  (Moran Rey, 1992) |
| 7. | Younger woman | 3  (Amine, al-Awadi, & Rabie, 1989; Brown et al., 2014; Gokceoglu & Kucukoglu, 2017)  1  U-shaped ((López M, Martínez G, & Zapata L, 2013; Rodrigo et al., 2019) | 0 | 1  (Duckett et al., 1998; Moran Rey, 1992) |
| 8. | Maternal ethnicity/race | 6  Jewish vs Arab women (Heldenberg, Tenenbaum, & Weizer, 1993)  Japanese vs. Caucasian women (Hla, Novotny, Kieffer, Mor, & Thiele, 2003)  Hispanic (vs. African American, White (Hurley, Black, Papas, & Quigg, 2008; Rozga, Kerver, & Olson, 2015)  Hispanic (vs. White) (R. Li et al., 2008)  Non-Caucasian (Kent et al., 2021) | 1  Non-Caucasian (Williams, Innis, Vogel, & Stephen, 1999) |  |
| 9. | Area of residence | 3  Rural vs. urban (Mosha, Laswai, & Dakiyo, 1998)  Japan vs. France/US (Negayama, Norimatsu, Barratt, & Bouville, 2012)  Outside US Midwest (Kirkland & Fein, 2003) | 0 | 1  City size (Moran Rey, 1992; Oommen, Vatsa, Paul, & Aggarwal, 2009)  Rural vs. urban (Zurayk & Shedid, 1981) |
| 10. | Female infant | 1  (Gokceoglu & Kucukoglu, 2017) | 0 | 0 |
| 11. | Younger infant age | 7  (Andrade Barcia & Valle Carrera, 1981; Frozzani, Zavoshi, & Azordeghan, 1992; López M et al., 2013; McCann, Baydar, & Williams, 2007; Schwartz et al., 2002; Sun, Chen, Yin, Wu, & Gao, 2017)  3 days pp (Wagner, Nommsen-Rivers, Chantry, & Dewey, 2013) | 4  (Karkee, Lee, Khanal, & Binns, 2014; Meirelles, Oliveira, Mello, Varela, & Fonseca, 2008; Perez-Escamilla, Segura-Millan, Pollitt, & Dewey, 1993; Williams et al., 1999) | 9  (Brown et al., 2014; Colin & Scott, 2002; Cooke, Sheehan, & Schmied, 2003; Essex, Smale, & Geddis, 1995; Flaherman, Beiler, Cabana, & Paul, 2016; Florack, Obermann-de Boer, van Kampen-Donker, van Wingen, & Kromhout, 1984; Tracz & Gajewska, 2020; Wang, Lau, Chow, & Chan, 2014; West, 1980; Yang et al., 2004) |
| 12. | SRIM across subsequent children | 0 | 0 | 1  (Whipps & Demirci, 2021) |
| 13. | Season of birth | Spring-summer (Forman et al., 1992) |  |  |
| **Psycho-social and Behavioral** |  |  |  |  |
| 14. | Breastfeeding beliefs and attitudes | 0 | 0 | 1  (Duckett et al., 1998) |
| 15. | Breastfeeding perceived control | 0 | 0 | 1  (Duckett et al., 1998) |
| 16. | Breastfeeding support from the child’s father | 0 | 1  (Hill & Aldag, 1991) | 0 |
| 17. | Lack of breastfeeding support from family | 3  (Chuang et al., 2007; Hill & Aldag, 1991; Rodrigo et al., 2019) | 0 | 0 |
| 18. | Prior BF experience | 0 | 1  BF as child (Segura-Millán et al., 1994) | 1  (Hill, Humenick, Argubright, & Aldag, 1997) |
| 19. | Breastfeeding intended duration | 0 | 1  (Huang, Lee, Huang, & Gau, 2009) | 0 |
| 20. | Maternal breastfeeding satisfaction | 0 | 2  (Cooke et al., 2003; Robert et al., 2014) | 0 |
| 21. | Maternal breastfeeding self-efficacy | 0 | 9  (Galipeau, Dumas, & Lepage, 2017; Gokceoglu & Kucukoglu, 2017; Gumussoy et al., 2020; Hill & Aldag, 1991; T. Li, Guo, Jiang, & Eldadah, 2019; McCarter-Spaulding & Kearney, 2001; Menekse et al., 2021; Otsuka, Dennis, Tatsuoka, & Jimba, 2008; Sandhi, Lee, Chipojola, Huda, & Kuo, 2020; Segura-Millán et al., 1994) | 0 |
| 22. | Maternal attachment | 0 | 1  (Gumussoy et al., 2020) | 0 |
| 23. | Breastfeeding convenience | 0 | 1  (Tully & Dewey, 1985) | 0 |
| 24. | Maternal depression and anxiety | 2  (Flaherman et al., 2016; Hazrat, Sumaira, & Shazia, 2017; Rodrigo et al., 2019) | 0 | 0 |
|  | Baby behaviors |  |  |  |
| 25. | -baby’s loss of interest in breastfeeding or breast refusal | 3  (Amir & Cwikel, 2005; Hill & Aldag, 1991; O'Sullivan, Perrine, & Rasmussen, 2015) | 0 | 0 |
| 26. | - perceived infant satisfaction | 0 | 4  (Cooke et al., 2003; Huang, Lee, et al., 2009; Moll Pons et al., 2012; Monteiro, Gomes, Stefanello, & Nakano, 2011) | 0 |
| 27. | - baby frequent feeding | 1  (Monteiro et al., 2011) | 0 | 0 |
| 28. | - infant irritability during feeds or crying/fussiness | 11  (Galipeau et al., 2017; Graffy, 1992; Hill & Aldag, 1991; McCann & Bender, 2006; Mohebati et al., 2021; Moll Pons et al., 2012; Monteiro et al., 2011; Perez-Escamilla, Mejia, & Dewey, 1992; Segura-Millán et al., 1994; Tully & Dewey, 1985; Wood, Sanders, Lewis, Woods, & Blackburn, 2017) | 0 | 0 |
| 29. | -infant suckling duration | 1  (Monteiro et al., 2011) | 0 | 1  (Hillervik-Lindquist, 1992) |
| 30. | -infant not suckling well or poor feeder | 4  (Galipeau et al., 2017; Hill & Aldag, 1991; Huang, Lee, et al., 2009; Mizuno, Fujimaki, & Sawada, 2004) | 0 | 0 |
| **Health care system** |  |  |  |  |
|  | Practices consistent with Ten Steps |  |  |  |
| 31. | Delivering in Baby Friendly Hospital (BFH) | 0 | 1  (Robert et al., 2014) | 0 |
| 32. | Health care professionals breastfeeding training |  |  |  |
| 33. | Timing of first breastfeeding | 3  (Lin, Lee, Yang, & Gau, 2011; Menekse et al., 2021; Sahin et al., 2013) | 0 | 1  (Hillervik-Lindquist, 1992) |
| 34. | Skin-to-skin | 0 | 1  (Sandhi et al., 2020) | 0 |
| 35. | Rooming-in | 0 | 1  (Perez-Escamilla et al., 1992) | 1  Without BF support (Perez-Escamilla et al., 1997) |
| 36. | Kangaroo Mother Care | 0 | 1  (Yilmaz, Kucukoglu, Aytekin Ozdemir, Ogul, & Aski, 2020) | 0 |
| 37. | Maternity ward formula supplementation | 4  (Huang, Gau, Huang, & Lee, 2009; Lin et al., 2011; Menekse et al., 2021; Tully & Dewey, 1985) | 0 | 0 |
| 38. | Prenatal BF support | 0 | 1  (Ume, Muhammad, & Muhammad Shahbaz Bakht, 2014) | 0 |
| 39. | Breastfeeding counseling and support from midwives, nurses, doulas | 0 | 8  (Blixt, Martensson, & Ekstrom, 2014; Chezem, Friesen, & Parker, 2004; Molinero Diaz, Burgos Rodriguez, & Mejia Ramirez de Arellano, 2015; Nommsen-Rivers, Mastergeorge, Hansen, Cullum, & Dewey, 2009; Silbert-Flagg, Busch, & Bataille, 2020; Vittoz et al., 2005) (Chezem et al., 2004)  Fathers’ BF education(Pisacane, Continisio, Aldinucci, D'Amora, & Continisio, 2005) | 1  (Vázquez Cancela et al., 2018) |
| 40. | Health care professional birth attendant | 1  Rural area  (Tai-Keun & Berlin, 1981) | 0 | 0 |
| 41. | Parenting programs | 0 | 0 | 1  (Lewkowitz et al., 2018) |
| 42. | e-health breastfeeding education | 0 | 0 | 1  Smartphone app (Lewkowitz et al., 2021) |
| 43. | Maternal dietary advise | 0 | 1  (Whichelow, 1979) | 1 |
| **Biomedical** |  |  |  |  |
| 44. | Hormonal contraceptives | 0 | 0 | 1  (Bryant et al., 2019) |
| 45. | Cesarean-section delivery | 6  (Bryant et al., 2019; Donmez & Korgali, 2021; Kent et al., 2021; Rodrigo et al., 2019; Sahin et al., 2013; Sun-Hee, 2019) | 0 | 1  (Hillervik-Lindquist, 1992) |
| 46. | Epidural anesthesia | 2  (Kent et al., 2021; Lin et al., 2011) | 0 | 0 |
| 47. | Maternal overweight or obesity | 1  Underweight and obesity (Guelinckx, Devlieger, Bogaerts, Pauwels, & Vansant, 2012)  6  Overweight/Obesity (Bryant et al., 2019; Jarlenski et al., 2014; Kair & Colaizy, 2016; Mallan, Daniels, Byrne, & de Jersey, 2018; Mok et al., 2008; O'Sullivan et al., 2015) | 0 | 0 |
| 48. | Poor mother’s health | 2  (Hill & Aldag, 1991; Rodrigo et al., 2019) | 0 | 0 |
| 49. | Less maternal sexual desire | 1  (Hillervik-Lindquist, 1992) | 0 | 0 |
| 50. | Maternal anemia | 1  (Henly et al., 1995) | 0 | 0 |
| 51. | Lower breast milk consumption | 1  (Hillervik-Lindquist, Hofvander, & Sjolin, 1991) | 0 | 0 |
| 52. | Newborn excessive weight loss or poor/less weight gain | 5  (Flaherman et al., 2016; Hill & Aldag, 1991; Hillerviklindquist, Hofvander, & Sjolin, 1991; Kent et al., 2021; Moll Pons et al., 2012; O'Sullivan et al., 2015) | 0 | 0 |
| 53. | Low birth weight, small-for-gestational age | 2  (Hill & Aldag, 1991; Tully & Dewey, 1985) | 0 | 1  (Hillervik-Lindquist, 1992) |
| 54. | Premature birth | 1  (Rodrigo et al., 2019) | 0 | 1 |
| 55. | Low infant urine output | 1  (Rodrigo et al., 2019) | 0 | 1 |
| **Breastfeeding knowledge, styles and problems** |  |  |  |  |
| 56. | Maternal breastfeeding knowledge | 0 | 0 | 1  (Duckett et al., 1998) |
| 57. | Not perceiving milk let down | 1  (Whichelow, 1979) | 0 | 0 |
| 58. | Delayed Onset of Lactation | 4  (Kent et al., 2021; Mohebati et al., 2021; O'Sullivan et al., 2015; Segura-Millán et al., 1994) | 0 | 0 |
| 59. | Early breastfeeding problems | 2  (Mohebati et al., 2021; O'Sullivan et al., 2015)  1  Sore nipples (Segura-Millán et al., 1994) | 0 | 0 |
| 60. | Breastmilk consumption test weighing | 0 | 1  (Kent, Hepworth, Langton, & Hartmann, 2015) | 0 |
| 61. | Nursing frequency | 0 | 2  (Lin et al., 2011; Sahin et al., 2013) | 0 |
| 62. | Introducing other milks or solids | 1  Replacing breast milk vs solids between breastfeeding episodes (Hillerviklindquist et al., 1991)  2  Mixed feeding (Huang, Lee, et al., 2009; Kent et al., 2021)  1  Early formula (Segura-Millán et al., 1994) | 0 | 0 |
| **Maternal lifestyles** |  |  |  |  |
| 63. | Specific foods consumption | 0 | 1  Pigs’ feet ((Kim et al., 2013) | 0 |
|  |  |  |  |  |

Amine, E. K., al-Awadi, F., & Rabie, M. (1989). Infant feeding pattern and weaning practices in Kuwait. *Journal of the Royal Society of Health, 109*(5), 178-180.

Amir, L. H., & Cwikel, J. (2005). Why do women stop breastfeeding? A closer look at 'not enough milk' among Israeli women in the Negev Region. *Breastfeeding review : professional publication of the Nursing Mothers' Association of Australia, 13*(3), 7-13.

Andrade Barcia, A., & Valle Carrera, E. (1981). [Breast feeding: causes for its discontinuation in 2 cities in Ecuador]. *Boletin de la Oficina Sanitaria Panamericana. Pan American Sanitary Bureau, 91*(5), 408-417.

Blixt, I., Martensson, L. B., & Ekstrom, A. C. (2014). Process-oriented training in breastfeeding for health professionals decreases women's experiences of breastfeeding challenges. *International breastfeeding journal, 9*(101251562), 15. doi: <https://dx.doi.org/10.1186/1746-4358-9-15>

Brown, C. R. L., Dodds, L., Legge, A., Bryanton, J., & Semenic, S. (2014). Factors influencing the reasons why mothers stop breastfeeding. *Canadian journal of public health = Revue canadienne de sante publique, 105*(3), e179-185.

Brownell, E., Howard, C. R., Dozier, A. M., & Lawrence, R. A. (2012). Delayed onset lactogenesis II predicts the cessation of any or exclusive breastfeeding. *Journal of Pediatrics, 161*(4), 608-614. doi: <http://dx.doi.org/10.1016/j.jpeds.2012.03.035>

Bryant, A. G., Bauer, A. E., Muddana, A., Wouk, K., Chetwynd, E., Yourkavitch, J., & Stuebe, A. M. (2019). The Lactational Effects of Contraceptive Hormones: an Evaluation (LECHE) Study. *Contraception, 100*(1), 48-53. doi: <https://dx.doi.org/10.1016/j.contraception.2019.03.040>

Chezem, J. C., Friesen, C. A., & Parker, C. G. (2004). Effect of Professional Postpartum Support on Infant Feeding Patterns Among Breastfeeding Participants in the WIC Program. *Family and Consumer Sciences Research Journal 32*(4), 349-360.

Chuang, C.-H., Chang, P.-J., Hsieh, W.-S., Guo, Y. L., Lin, S.-H., Lin, S.-J., & Chen, P.-C. (2007). The combined effect of employment status and transcultural marriage on breast feeding: a population-based survey in Taiwan. *Paediatric and perinatal epidemiology, 21*(4), 319-329.

Colin, W. B., & Scott, J. A. (2002). Breastfeeding: reasons for starting, reasons for stopping and problems along the way. *Breastfeeding review : professional publication of the Nursing Mothers' Association of Australia, 10*(2), 13-19.

Cooke, M., Sheehan, A., & Schmied, V. (2003). A description of the relationship between breastfeeding experiences, breastfeeding satisfaction, and weaning in the first 3 months after birth. *Journal of human lactation : official journal of International Lactation Consultant Association, 19*(2), 145-156.

Donmez, A. Y., & Korgali, E. U. (2021). The relationship between the perception of breast milk of parents with term infants and exclusive breastfeeding in the postnatal first six months. *TURKISH ARCHIVES OF PEDIATRICS, 56*(2), 164-172. doi: 10.14744/TurkPediatriArs.2020.75875

Duckett, L., Henly, S., Avery, M., Potter, S., Hills-Bonczyk, S., Hulden, R., & Savik, K. (1998). A theory of planned behavior-based structural model for breast-feeding. *Nursing research, 47*(6), 325-336.

Einterz, E. M., & Bates, M. E. (1994). Early childhood feeding practices in northern Cameroon. *Transactions of the Royal Society of Tropical Medicine and Hygiene, 88*(5), 575-576.

Essex, C., Smale, P., & Geddis, D. (1995). Breastfeeding rates in New Zealand in the first 6 months and the reasons for stopping. *The New Zealand medical journal, 108*(1007), 355-357.

Flaherman, V. J., Beiler, J. S., Cabana, M. D., & Paul, I. M. (2016). Relationship of newborn weight loss to milk supply concern and anxiety: the impact on breastfeeding duration. *Maternal & child nutrition, 12*(3), 463-472. doi: <https://dx.doi.org/10.1111/mcn.12171>

Florack, E., Obermann-de Boer, G., van Kampen-Donker, M., van Wingen, J., & Kromhout, D. (1984). Breast-feeding, bottle-feeding and related factors. The Leiden Pre-School Children Study. *Acta paediatrica Scandinavica, 73*(6), 789-795.

Forman, M. R., Lewando-Hundt, G., Graubard, B. I., Chang, D., Sarov, B., Naggan, L., & Berendes, H. W. (1992). Factors influencing milk insufficiency and its long-term health effects: the Bedouin Infant Feeding Study. *International journal of epidemiology, 21*(1), 53-58.

Frozzani, M. D., Zavoshi, R., & Azordeghan, F. (1992). Duration and cases of cessation of breast feeding in working mothers in Ghazvin, Iran (Vol. 6, pp. 93-95).

Galipeau, R., Dumas, L., & Lepage, M. (2017). Perception of Not Having Enough Milk and Actual Milk Production of First-Time Breastfeeding Mothers: Is There a Difference? *Breastfeeding medicine : the official journal of the Academy of Breastfeeding Medicine, 12*(101260777), 210-217. doi: <https://dx.doi.org/10.1089/bfm.2016.0183>

Gokceoglu, E., & Kucukoglu, S. (2017). The relationship between insufficient milk perception and breastfeeding self-efficacy among Turkish mothers. *Global health promotion, 24*(4), 53-61. doi: <https://dx.doi.org/10.1177/1757975916635080>

Graffy, J. P. (1992). Mothers' attitudes to and experience of breast feeding: a primary care study. *The British journal of general practice : the journal of the Royal College of General Practitioners, 42*(355), 61-64.

Guelinckx, I., Devlieger, R., Bogaerts, A., Pauwels, S., & Vansant, G. (2012). The effect of pre-pregnancy BMI on intention, initiation and duration of breast-feeding. *Public health nutrition, 15*(5), 840-848. doi: <https://dx.doi.org/10.1017/S1368980011002667>

Gumussoy, S., Celik, N. A., Guner, O., Kiratli, D., Atan, S. U., & Kavlak, O. (2020). Investigation of the Relationship Between Maternal Attachment and Breastfeeding Self-Efficacy and Affecting Factors in Turkish Sample. *Journal of pediatric nursing, 54*(jns, 8607529), e53-e60. doi: <https://dx.doi.org/10.1016/j.pedn.2020.04.022>

Hazrat, A., Sumaira, H., & Shazia, S. (2017). Perceptions of insufficient breast milk: a comparison of depressed and non-depressed lactating mothers (Vol. 67, pp. 226-231).

Heldenberg, D., Tenenbaum, G., & Weizer, S. (1993). Breast-feeding habits among Jewish and Arab mothers in Hadera County, Israel. *Journal of pediatric gastroenterology and nutrition, 17*(1), 86-91.

Henly, S. J., Anderson, C. M., Avery, M. D., Hills-Bonczyk, S. G., Potter, S., & Duckett, L. J. (1995). Anemia and insufficient milk in first-time mothers. *Birth (Berkeley, Calif.), 22*(2), 86-92.

Herrera, M. (2008). Milk insufficiency in Esmeraldas, Ecuador: A multidisciplinary approach. *Dissertation Abstracts International Section A: Humanities and Social Sciences, 69*(5-A), 1858.

Hill, P. D., & Aldag, J. (1991). Potential indicators of insufficient milk supply syndrome. *Research in nursing & health, 14*(1), 11-19.

Hill, P. D., Humenick, S. S., Argubright, T. M., & Aldag, J. C. (1997). Effects of parity and weaning practices on breastfeeding duration. *Public health nursing (Boston, Mass.), 14*(4), 227-234.

Hillervik-Lindquist, C. (1992). Studies on perceived breast-milk insufficiency: relation to attitude and practice. *Journal of Biosocial Science, 24*(3), 413-425.

Hillervik-Lindquist, C., Hofvander, Y., & Sjolin, S. (1991). Studies on perceived breast milk insufficiency. III. Consequences for breast milk consumption and growth. *Acta paediatrica Scandinavica, 80*(3), 297-303.

Hillerviklindquist, C., Hofvander, Y., & Sjolin, S. (1991). STUDIES ON PERCEIVED BREAST-MILK INSUFFICIENCY - RELATIONSHIP TO THE WEANING PROCESS. *JOURNAL OF HUMAN NUTRITION AND DIETETICS, 4*(5), 317-326. doi: 10.1111/j.1365-277X.1991.tb00113.x

Hla, M. M., Novotny, R., Kieffer, E. C., Mor, J., & Thiele, M. (2003). Early weaning among Japanese women in Hawaii. *Journal of Biosocial Science, 35*(2), 227-241. doi: <http://dx.doi.org/10.1017/S002193200300227X>

Huang, Y., Gau, M. L., Huang, C. M., & Lee, J. T. (2009). Supplementation with cup-feeding as a substitute for bottle-feeding to promote breastfeeding. *Chang Gung medical journal, 32*(4), 423-431.

Huang, Y., Lee, J. T., Huang, C. M., & Gau, M. L. (2009). Factors related to maternal perception of milk supply while in the hospital. *The Journal of Nursing Research, 17*(3), 179-188. doi: <https://dx.doi.org/10.1097/JNR.0b013e3181b25558>

Hurley, K. M., Black, M. M., Papas, M. A., & Quigg, A. M. (2008). Variation in breastfeeding behaviours, perceptions, and experiences by race/ethnicity among a low-income statewide sample of Special Supplemental Nutrition Program for Women, Infants, and Children (WIC) participants in the United States. *MATERNAL AND CHILD NUTRITION, 4*(2), 95-105. doi: 10.1111/j.1740-8709.2007.00105.x

Jarlenski, M., McManus, J., Diener-West, M., Schwarz, E. B., Yeung, E., & Bennett, W. L. (2014). Association between support from a health professional and breastfeeding knowledge and practices among obese women: evidence from the Infant Practices Study II. *Women's health issues : official publication of the Jacobs Institute of Women's Health, 24*(6), 641-648. doi: <https://dx.doi.org/10.1016/j.whi.2014.08.002>

Kair, L. R., & Colaizy, T. T. (2016). When Breast Milk Alone Is Not Enough: Barriers to Breastfeeding Continuation among Overweight and Obese Mothers. *Journal of human lactation : official journal of International Lactation Consultant Association, 32*(2), 250-257. doi: <https://dx.doi.org/10.1177/0890334415605303>

Karkee, R., Lee, A. H., Khanal, V., & Binns, C. W. (2014). Infant feeding information, attitudes and practices: a longitudinal survey in central Nepal. *International breastfeeding journal, 9*(101251562), 14. doi: <https://dx.doi.org/10.1186/1746-4358-9-14>

Keemer, F. (2013). Breastfeeding self-efficacy of women using second-line strategies for healthy term infants in the first week postpartum: an Australian observational study. *International breastfeeding journal, 8*(1), 18. doi: <https://dx.doi.org/10.1186/1746-4358-8-18>

Kent, J. C., Ashton, E., Hardwick, C. M., Alethea, R., Murray, K. M., & Geddes, D. T. (2021). Causes of perception of insufficient milk supply in Western Australian mothers. *Maternal & child nutrition, 17*(1), e13080. doi: <https://dx.doi.org/10.1111/mcn.13080>

Kent, J. C., Hepworth, A. R., Langton, D. B., & Hartmann, P. E. (2015). Impact of Measuring Milk Production by Test Weighing on Breastfeeding Confidence in Mothers of Term Infants. *Breastfeeding medicine : the official journal of the Academy of Breastfeeding Medicine, 10*(6), 318-325. doi: <https://dx.doi.org/10.1089/bfm.2015.0025>

Kim, M.-K., Shin, J.-S., Patel, R. A., Min, Y. S., Song, H. J., Sohn, U. D., . . . Kim, D.-S. (2013). The effects of pigs' feet consumption on lactation. *ECOLOGY OF FOOD AND NUTRITION, 52*(3), 223-238. doi: <https://dx.doi.org/10.1080/03670244.2012.706157>

Kirkland, V. L., & Fein, S. B. (2003). Characterizing reasons for breastfeeding cessation throughout the first year postpartum using the construct of thriving. *Journal of human lactation : official journal of International Lactation Consultant Association, 19*(3), 278-285.

Lewkowitz, A. K., Lopez, J. D., Stein, R. I., Rhoades, J. S., Schulz, R. C., Woolfolk, C. L., . . . Cahill, A. G. (2018). Effect of a Home-Based Lifestyle Intervention on Breastfeeding Initiation Among Socioeconomically Disadvantaged African American Women with Overweight or Obesity. *Breastfeeding medicine : the official journal of the Academy of Breastfeeding Medicine, 13*(6), 418-425. doi: <https://dx.doi.org/10.1089/bfm.2018.0006>

Lewkowitz, A. K., Werner, E. F., Rouse, D. J., Lopez, J. D., Ranney, M. L., Cahill, A. G., . . . Savitz, D. A. (2021). Effect of a Novel Smartphone Application on Breastfeeding Rates among Low-Income, First-Time Mothers Intending to Exclusively Breastfeed: Secondary Analysis of a Randomized Controlled Trial. *Breastfeeding Medicine, 16*(1), 59-67. doi: <http://dx.doi.org/10.1089/bfm.2020.0240>

Li, R., Fein, S. B., Chen, J., & Grummer-Strawn, L. M. (2008). Why mothers stop breastfeeding: mothers' self-reported reasons for stopping during the first year. *Pediatrics, 122 Suppl 2*(oxv, 0376422), S69-76. doi: <https://dx.doi.org/10.1542/peds.2008-1315i>

Li, T., Guo, N., Jiang, H., & Eldadah, M. (2019). Breastfeeding Self-Efficacy Among Parturient Women in Shanghai: A Cross-Sectional Study. *Journal of human lactation : official journal of International Lactation Consultant Association, 35*(3), 583-591. doi: <https://dx.doi.org/10.1177/0890334418812044>

Lin, S.-Y., Lee, J.-T., Yang, C.-C., & Gau, M.-L. (2011). Factors related to milk supply perception in women who underwent cesarean section. *The journal of nursing research : JNR, 19*(2), 94-101. doi: <https://dx.doi.org/10.1097/JNR.0b013e31821988e9>

López M, B. E., Martínez G, L., & Zapata L, N. J. (2013). Motivos del abandono temprano de la lactancia materna exclusiva: un problema de salud pública no resuelto en la ciudad de Medellín. *Rev. Fac. Nac. Salud Pública, 31*(1), 117-126.

Mallan, K. M., Daniels, L. A., Byrne, R., & de Jersey, S. J. (2018). Comparing barriers to breastfeeding success in the first month for non-overweight and overweight women. *BMC pregnancy and childbirth, 18*(1), 461. doi: <https://dx.doi.org/10.1186/s12884-018-2094-5>

McCann, M. F., Baydar, N., & Williams, R. L. (2007). Breastfeeding attitudes and reported problems in a national sample of WIC participants. *Journal of human lactation : official journal of International Lactation Consultant Association, 23*(4), 314-324.

McCann, M. F., & Bender, D. E. (2006). Perceived insufficient milk as a barrier to optimal infant feeding: examples from Bolivia. *Journal of Biosocial Science, 38*(3), 341-364.

McCarter-Spaulding, D. E., & Kearney, M. H. (2001). Parenting self-efficacy and perception of insufficient breast milk. *Journal of obstetric, gynecologic, and neonatal nursing : JOGNN, 30*(5), 515-522.

Meirelles, C. d. A. B., Oliveira, M. I. d. C., Mello, R. R. d., Varela, M. A. B., & Fonseca, V. d. M. (2008). Justificativas para uso de suplemento em recém-nascidos de baixo risco de um Hospital Amigo da Criança. *Cad. saúde pública, 24*(9), 2001-2012.

Menekse, D., Tiryaki, O., Karakaya Suzan, O., & Cinar, N. (2021). An investigation of the relationship between mother's personality traits, breastfeeding self-efficacy, and perception of insufficient milk supply. *Health care for women international*, 1-17. doi: <https://dx.doi.org/10.1080/07399332.2021.1892114>

Mizuno, K., Fujimaki, K., & Sawada, M. (2004). Sucking behavior at breast during the early newborn period affects later breast-feeding rate and duration of breast-feeding. *Pediatrics international : official journal of the Japan Pediatric Society, 46*(1), 15-20.

Mohebati, L. M., Hilpert, P., Bath, S., Rayman, M. P., Raats, M. M., Martinez, H., & Caulfield, L. E. (2021). Perceived insufficient milk among primiparous, fully breastfeeding women: Is infant crying important? *Matern Child Nutr, 17*(3), e13133. doi: 10.1111/mcn.13133

Mok, E., Multon, C., Piguel, L., Barroso, E., Goua, V., Christin, P., . . . Hankard, R. (2008). Decreased full breastfeeding, altered practices, perceptions, and infant weight change of prepregnant obese women: a need for extra support. *Pediatrics, 121*(5), e1319-1324. doi: <https://dx.doi.org/10.1542/peds.2007-2747>

Molinero Diaz, P., Burgos Rodriguez, M. J., & Mejia Ramirez de Arellano, M. (2015). [Results of a health education intervention in the continuity of breastfeeding]. *Enfermeria clinica, 25*(5), 232-238. doi: <https://dx.doi.org/10.1016/j.enfcli.2015.05.002>

Moll Pons, J. M., Prieto Valle, J. M., Sanchez Martinez, A., Lopez Leon, M. P., Arana Galan, J. M., & Frontera Juan, G. (2012). Prevalence of breastfeeding in the health sector Hospital Son Llatzer (Palma de Mallorca). *Acta Pediatrica Espanola, 70*(5), 186-194.

Monteiro, J. C. D., Gomes, F. A., Stefanello, J., & Nakano, A. M. S. (2011). WOMEN'S PERCEPTIONS ON MILK PRODUCED AND CHILD SATISFACTION DURING EXCLUSIVE BREAST FEEDING. *TEXTO & CONTEXTO ENFERMAGEM, 20*(2), 359-367.

Moran Rey, J. (1992). [Breast feeding in Spain. Current situation]. *Anales espanoles de pediatria, 36*(1), 45-50.

Mosha, T. C., Laswai, H. S., & Dakiyo, S. O. S. (1998). Breastfeeding, weaning practices and anthropometric status of children in Morogoro district, Tanzania. *ECOLOGY OF FOOD AND NUTRITION, 37*(4), 309-338. doi: 10.1080/03670244.1998.9991551

Negayama, K., Norimatsu, H., Barratt, M., & Bouville, J.-F. (2012). Japan-France-US comparison of infant weaning from mother's viewpoint. *Journal of reproductive and infant psychology, 30*(1), 77-91.

Nommsen-Rivers, L. A., Mastergeorge, A. M., Hansen, R. L., Cullum, A. S., & Dewey, K. G. (2009). Doula care, early breastfeeding outcomes, and breastfeeding status at 6 weeks postpartum among low-income primiparae. *Journal of obstetric, gynecologic, and neonatal nursing : JOGNN, 38*(2), 157-173. doi: <https://dx.doi.org/10.1111/j.1552-6909.2009.01005.x>

O'Sullivan, E. J., Perrine, C. G., & Rasmussen, K. M. (2015). Early Breastfeeding Problems Mediate the Negative Association between Maternal Obesity and Exclusive Breastfeeding at 1 and 2 Months Postpartum. *The Journal of nutrition, 145*(10), 2369-2378. doi: <https://dx.doi.org/10.3945/jn.115.214619>

Oommen, A., Vatsa, M., Paul, V. K., & Aggarwal, R. (2009). Breastfeeding practices of urban and rural mothers. *Indian pediatrics, 46*(10), 891-894.

Otsuka, K., Dennis, C.-L., Tatsuoka, H., & Jimba, M. (2008). The relationship between breastfeeding self-efficacy and perceived insufficient milk among Japanese mothers. *Journal of obstetric, gynecologic, and neonatal nursing : JOGNN, 37*(5), 546-555. doi: <https://dx.doi.org/10.1111/j.1552-6909.2008.00277.x>

Perez-Escamilla, R., Lutter, C. K., Wickham, C., Phillips, M., TrevinoSiller, S., & Sanghvi, T. (1997). Identification of risk factors for short breastfeeding duration in Mexico City through survival analysis. *ECOLOGY OF FOOD AND NUTRITION, 36*(1), 43-64. doi: 10.1080/03670244.1997.9991504

Perez-Escamilla, R., Mejia, L. A., & Dewey, K. G. (1992). NEONATAL FEEDING PATTERNS AND REPORTS OF INSUFFICIENT MILK AMONG LOW-INCOME URBAN MEXICAN MOTHERS. *ECOLOGY OF FOOD AND NUTRITION, 27*(2), 91-102. doi: 10.1080/03670244.1992.9991231

Perez-Escamilla, R., Segura-Millan, S., Pollitt, E., & Dewey, K. G. (1993). Determinants of lactation performance across time in an urban population from Mexico. *Social science & medicine (1982), 37*(8), 1069-1078.

Pisacane, A., Continisio, G. I., Aldinucci, M., D'Amora, S., & Continisio, P. (2005). A controlled trial of the father's role in breastfeeding promotion. *Pediatrics, 116*(4), e494-498.

Robert, E., Coppieters, Y., Swennen, B., & Dramaix, M. (2014). The Reasons for Early Weaning, Perceived Insufficient Breast Milk, and Maternal Dissatisfaction: Comparative Studies in Two Belgian Regions. *International scholarly research notices, 2014*(101641951), 678564. doi: <https://dx.doi.org/10.1155/2014/678564>

Rodrigo, R., Rodrigo, A., Liyanage, N., Hatahagoda, W., & Hewavitharana, U. (2019). Maternal Perception of Adequacy of Mother's Milk Among Mothers Giving Birth at a Teaching Hospital in Sri Lanka. *Journal of human lactation : official journal of International Lactation Consultant Association, 35*(1), 171-180. doi: <https://dx.doi.org/10.1177/0890334418773304>

Rozga, M. R., Kerver, J. M., & Olson, B. H. (2015). Self-reported reasons for breastfeeding cessation among low-income women enrolled in a peer counseling breastfeeding support program. *Journal of human lactation : official journal of International Lactation Consultant Association, 31*(1), 129-190. doi: <https://dx.doi.org/10.1177/0890334414548070>

Sahin, H., Yilmaz, M., Aykut, M., Balci, E., Sagiroglu, M., & Ozturk, A. (2013). Risk factors for breastfeeding problems in mothers applied to two public healthcare centers in Kayseri province. *Turk Pediatri Arsivi, 48*(2), 145-151. doi: <http://dx.doi.org/10.4274/tpa.1034>

Sandhi, A., Lee, G. T., Chipojola, R., Huda, M. H., & Kuo, S.-Y. (2020). The relationship between perceived milk supply and exclusive breastfeeding during the first six months postpartum: a cross-sectional study. *International breastfeeding journal, 15*(1), 65. doi: <https://dx.doi.org/10.1186/s13006-020-00310-y>

Schwartz, K., D'Arcy, H. J. S., Gillespie, B., Bobo, J., Longeway, M., & Foxman, B. (2002). Factors associated with weaning in the first 3 months postpartum. *The Journal of family practice, 51*(5), 439-444.

Segura-Millán, S., Dewey, K. G., & Perez-Escamilla, R. (1994). Factors associated with perceived insufficient milk in a low-income urban population in Mexico. *J Nutr, 124*(2), 202-212. doi: 10.1093/jn/124.2.202

Silbert-Flagg, J., Busch, D., & Bataille, J. A. (2020). Mothers' Perceptions of the Influence of a Breastfeeding Support Group in Achieving Their Breastfeeding Goals. *CLINICAL LACTATION, 11*(2), 74-83. doi: 10.1891/CLINLACT-D-19-00019

Sun-Hee, K. I. M. (2019). The Breast Feeding Adaptation Scale-Short Form: Development and Testing of Its Psychometric Properties and Measurement Invariance. *Child Health Nursing Research*, 333-343.

Sun, K., Chen, M., Yin, Y., Wu, L., & Gao, L. (2017). Why Chinese mothers stop breastfeeding: Mothers' self-reported reasons for stopping during the first six months. *Journal of child health care : for professionals working with children in the hospital and community, 21*(3), 353-363. doi: <https://dx.doi.org/10.1177/1367493517719160>

Tai-Keun, P., & Berlin, P. (1981). Prevalence of Exclusive and Extended Breastfeeding Among Rural Korean Women. *Yonsei Medical Journal*, 108-121.

Tracz, J., & Gajewska, D. (2020). Factors Influencing the Duration of Breastfeeding among Polish Women. *Journal of mother and child, 24*(1), 39-46. doi: <https://dx.doi.org/10.34763/jmotherandchild.2020241.2006.000007>

Tully, J., & Dewey, K. G. (1985). Private fears, global loss: a cross-cultural study of the insufficient milk syndrome. *Med Anthropol, 9*(3), 225-243. doi: 10.1080/01459740.1985.9965934

Ume, S., Muhammad, I., & Muhammad Shahbaz Bakht, K. (2014). &lt;A&gt; comparative study on mothers in relation to antenatal counseling and other factors affecting their breastfeeding practices (Vol. 6, pp. 166-168).

Vázquez Cancela, L., Estany Gestal, A., Anido Rodríguez, M. C., Conde Vida, M. J., Folgosa Rodríguez, M. S., González Varela, M. P., . . . Varela Castiñeira, M. C. (2018). Efectividad de un taller formativo en lactancia materna exclusiva. *Metas enferm, 21*(4), 15-21.

Vittoz, J.-P., Francois, P., Pons, J.-C., Gelbert-Baudino, N., Ayral, A.-S., Duc, C., . . . Labarere, J. (2005). Efficacy of breastfeeding support provided by trained clinicians during an early, routine, preventive visit: A prospective, randomized, open trial of 226 mother-infant pairs. *Pediatrics, 115*(2), e139-e146. doi: <http://dx.doi.org/10.1542/peds.2004-1362>

Wagner, E. A., Nommsen-Rivers, L. A., Chantry, C. J., & Dewey, K. G. (2013). Breastfeeding concerns at 3 and 7 days postpartum and feeding status at 2 months. *Pediatrics, 132*(4), e865-e875. doi: <http://dx.doi.org/10.1542/peds.2013-0724>

Wang, W., Lau, Y., Chow, A., & Chan, K. S. (2014). Breast-feeding intention, initiation and duration among Hong Kong Chinese women: a prospective longitudinal study. *Midwifery, 30*(6), 678-687. doi: <https://dx.doi.org/10.1016/j.midw.2013.07.015>

West, C. P. (1980). Factors influencing the duration of breast-feeding. *Journal of Biosocial Science, 12*(3), 325-331.

Whichelow, M. J. (1979). Breast feeding in Cambridge, England: factors affecting the mother's milk supply. *Journal of advanced nursing, 4*(3), 253-261.

Whipps, M. D., & Demirci, J. R. (2021). The sleeper effect of perceived insufficient milk supply in US mothers. *Public health nutrition, 24*(5), 935-941. doi: <https://dx.doi.org/10.1017/S1368980020001482>

Williams, P. L., Innis, S. M., Vogel, A. M., & Stephen, L. J. (1999). Factors influencing infant feeding practices of mothers in Vancouver. *Canadian journal of public health = Revue canadienne de sante publique, 90*(2), 114-119.

Wood, N. K., Sanders, E. A., Lewis, F. M., Woods, N. F., & Blackburn, S. T. (2017). Pilot test of a home-based program to prevent perceived insufficient milk. *Women and birth : journal of the Australian College of Midwives, 30*(6), 472-480. doi: <https://dx.doi.org/10.1016/j.wombi.2017.04.006>

Yang, Q., Wen, S. W., Dubois, L., Chen, Y., Walker, M. C., & Krewski, D. (2004). Determinants of breast-feeding and weaning in Alberta, Canada. *Journal of obstetrics and gynaecology Canada : JOGC = Journal d'obstetrique et gynecologie du Canada : JOGC, 26*(11), 975-981.

Yilmaz, F., Kucukoglu, S., Aytekin Ozdemir, A., Ogul, T., & Aski, N. (2020). The Effect of Kangaroo Mother Care, Provided in the Early Postpartum Period, on the Breastfeeding Self-Efficacy Level of Mothers and the Perceived Insufficient Milk Supply. *The Journal of perinatal & neonatal nursing, 34*(1), 80-87. doi: <https://dx.doi.org/10.1097/JPN.0000000000000434>

Zurayk, H. C., & Shedid, H. E. (1981). The trend away from breast feeding in a developing country. A women's perspective. *Journal of Tropical Pediatrics, 27*(5), 237-244.
