# Supplementary material for: Risk factors for self‐reported insufficient milk during the first 6 months of life: A systematic review
Source: Matern Child Nutr. 2022 Mar 28;18(Suppl 3):e13353. doi: 10.1111/mcn.13353 (PMC9113468; doi:10.1111/mcn.13353)
Supplement: Supplementary file 2 — Supporting Information. [file MCN-18-e13353-s001.docx]

**Online Appendix B- Delayed Onset of Lactation Risk Factors**

| **Delayed Onset of Lactation Risk Factors. Systematic Review.** | | | | |
| --- | --- | --- | --- | --- |
| Domain | Factors | # of studies | | |
|  |  | Risk | Protection | Neutral |
| **Socio-economic** |  |  |  |  |
| 1. | Poverty, low household income | 2  (Brownell, Howard, Dozier, & Lawrence, 2012; Haile, Chavan, Teweldeberhan, & Chertok, 2017) | 0 | 0 |
| 2. | Maternal employment | 1  (Brownell et al., 2012) | 0 | 0 |
| **Demographic** |  |  |  |  |
| 3. | Primiparity | 5  Risk factor (Brownell et al., 2012; Huang et al., 2020; Scott, Binns, & Oddy, 2007)  Only among primiparous pumping BM after C-section(Chapman & Perez-Escamilla, 2000)  Primiparous delivering large infants (Dewey, Nommsen-Rivers, Heinig, & Cohen, 2003a) | 0 | 0 |
| 4. | Higher maternal age | 3  (Nommsen-Rivers, Chantry, Peerson, Cohen, & Dewey, 2010; Preusting, Brumley, Odibo, Spatz, & Louis, 2017; Rocha et al., 2020) | 0 | 0 |
| **Psycho-social and Behavioral** |  |  |  |  |
| 5. | Maternal anxiety stress and depression | 2  (Flaherman, Beiler, Cabana, & Paul, 2016; Rocha et al., 2020) | 0 | 0 |
| 6. | Maternal responsiveness to infant crying | 0 | 1  (Mohebati et al., 2021) | 0 |
| **Health care system** |  |  |  |  |
|  | Maternity practices consistent with Ten Steps |  |  |  |
| 7. | Early introduction of CMF | 4  (Hruschka, Sellen, Stein, & Martorell, 2003; Huang et al., 2020; Nommsen-Rivers, Mastergeorge, Hansen, Cullum, & Dewey, 2009; Rocha et al., 2020) | 0 | 0 |
| 8. | Timely BF initiation | 0 | 1  (Chapman & Perez-Escamilla, 2000) | 0 |
| 9. | Rooming-in | 0 | 1  (Brownell et al., 2012) | 0 |
| 10. | Breastfeeding counseling and support from providers | 0 | 1  (Brownell et al., 2012) | 0 |
| **Biomedical** |  |  |  |  |
| 11. | Cesarean-section delivery | 3  (Dewey et al., 2003a; Haile et al., 2017)  unscheduled (Chapman & Perez-Escamilla, 2000) | 0 | 0 |
| 12. | Gestational diabetes | 1  (Chertok & Sherby, 2016) | 0 | 0 |
| 13. | Maternal overweight or obesity | 4  (Dewey, Nommsen-Rivers, Heinig, & Cohen, 2003b; Nommsen-Rivers et al., 2010; Preusting et al., 2017; Sellen, Thompson, Hruschka, Stein, & Martorell, 2004) | 0 | 1  (Mallan, Daniels, Byrne, & de Jersey, 2018) |
| 14. | Excessive gestational weight gain | 4  (3)  (Haile et al., 2017; Huang et al., 2019; Huang et al., 2020; Preusting et al., 2017)  (1)  Among non-Hispanic White (Haile et al., 2017) | 0 | 0 |
| 15. | Epidural anesthesia/pain medication | 3  (Brownell et al., 2012; Haile et al., 2017; Preusting et al., 2017) | 0 | 0 |
| 16. | Prolonged stage II labor duration | 3  (Chapman & Pérez-Escamilla, 1999; Dewey et al., 2003b; Kung & Bajorek, 2008) | 0 | 0 |
| 17. | Hormonal contraceptives | 0 | 0 | 1  (Turok et al., 2017) |
| 18. | Excessive newborn weight loss | 2  (Dewey et al., 2003a; Flaherman et al., 2016) | 0 | 0 |
| 19. | Low birth weight, small-for-gestational age | 0 | 1  (Huang et al., 2020) | 0 |
| 20. | Large birth weight | 1  (Nommsen-Rivers et al., 2010) | 0 | 0 |
| 21. | Gestational age | 1  (Huang et al., 2020) | 0 | 0 |
| 22. | Lower Apgar score | 1  (Matias, Nommsen-Rivers, Creed-Kanashiro, & Dewey, 2010) | 0 | 0 |
| **Breastfeeding knowledge, styles and problems** |  |  |  |  |
| 23. | Lack of BF experience | 1  (Brownell et al., 2012) | 0 | 0 |
| 24. | Pacifier | 0 | 1  (Nommsen-Rivers et al., 2009) | 0 |
| 25. | Breastmilk expression/pumping | 1  among primiparae (Chapman & Perez-Escamilla, 2000) | 1  (Fok et al., 2019) | 0 |
| 26. | Nursing frequency/ on-demand BF | 0 | 2  (Brownell et al., 2012; Nommsen-Rivers et al., 2010) | 1 |
| 27. | Early breastfeeding problems | 1  (Dewey et al., 2003b) | 1  Nipple discomfort (Nommsen-Rivers et al., 2010) | 0 |
| 28. | Poor latch | 1  (Huang et al., 2020) | 0 | 0 |
| **Maternal lifestyles** |  |  |  |  |
| 29. | Adequate maternal sleep | 0 | 1  (Casey et al., 2019) | 0 |
| 30. | Tobbaco use | (Brownell et al., 2012) |  |  |
| 31. | Alcohol during pregnancy | 1  (Rocha et al., 2020) | 0 | 0 |

Brownell, E., Howard, C. R., Dozier, A. M., & Lawrence, R. A. (2012). Delayed onset lactogenesis II predicts the cessation of any or exclusive breastfeeding. *Journal of Pediatrics, 161*(4), 608-614. doi: <http://dx.doi.org/10.1016/j.jpeds.2012.03.035>

Casey, T., Crodian, J., Cummings, S., Plaut, K., Sun, H., Burgess, H. J., . . . Ahmed, A. (2019). Delayed Lactogenesis II is Associated With Lower Sleep Efficiency and Greater Variation in Nightly Sleep Duration in the Third Trimester. *Journal of human lactation : official journal of International Lactation Consultant Association, 35*(4), 713-724. doi: <http://dx.doi.org/10.1177/0890334419830991>

Chapman, D. J., & Perez-Escamilla, R. (2000). Maternal perception of the onset of lactation is a valid, public health indicator of lactogenesis stage II. *The Journal of nutrition, 130*(12), 2972-2980.

Chapman, D. J., & Pérez-Escamilla, R. (1999). Identification of risk factors for delayed onset of lactation. *J Am Diet Assoc, 99*(4), 450-454; quiz 455-456. doi: 10.1016/s0002-8223(99)00109-1

Chertok, I. R., & Sherby, E. (2016). Breastfeeding Self-efficacy of Women With and Without Gestational Diabetes. *MCN Am J Matern Child Nurs, 41*(3), 173-178. doi: 10.1097/nmc.0000000000000233

Dewey, K. G., Nommsen-Rivers, L. A., Heinig, M. J., & Cohen, R. J. (2003a). Risk factors for suboptimal infant breastfeeding behavior, delayed onset of lactation, and excess neonatal weight loss. *Pediatrics, 112*(3 I), 607-619. doi: <http://dx.doi.org/10.1542/peds.112.3.607>

Dewey, K. G., Nommsen-Rivers, L. A., Heinig, M. J., & Cohen, R. J. (2003b). Risk factors for suboptimal infant breastfeeding behavior, delayed onset of lactation, and excess neonatal weight loss. *Pediatrics, 112*(3 Pt 1), 607-619. doi: 10.1542/peds.112.3.607

Flaherman, V. J., Beiler, J. S., Cabana, M. D., & Paul, I. M. (2016). Relationship of newborn weight loss to milk supply concern and anxiety: the impact on breastfeeding duration. *Maternal & child nutrition, 12*(3), 463-472. doi: <https://dx.doi.org/10.1111/mcn.12171>

Fok, D., Aris, I. M., Ho, J., Chan, Y.-H., Rauff, M., Lui, J. K. C., . . . Mattar, C. N. Z. (2019). Early initiation and regular breast milk expression reduces risk of lactogenesis II delay in at-risk Singaporean mothers in a randomised trial. *Singapore medical journal, 60*(2), 80-88. doi: <https://dx.doi.org/10.11622/smedj.2018067>

Haile, Z. T., Chavan, B. B., Teweldeberhan, A., & Chertok, I. R. (2017). Association Between Gestational Weight Gain and Delayed Onset of Lactation: The Moderating Effects of Race/Ethnicity. *Breastfeeding medicine : the official journal of the Academy of Breastfeeding Medicine, 12*(101260777), 79-85. doi: <https://dx.doi.org/10.1089/bfm.2016.0134>

Hruschka, D. J., Sellen, D. W., Stein, A. D., & Martorell, R. (2003). Delayed onset of lactation and risk of ending full breast-feeding early in rural Guatemala. *The Journal of nutrition, 133*(8), 2592-2599.

Huang, L., Chen, X., Zhang, Y., Zhong, C., Li, Q., Li, X., . . . Wang, W. (2019). Gestational weight gain is associated with delayed onset of lactogenesis in the TMCHC study: A prospective cohort study. *Clinical Nutrition, 38*(5), 2436-2441. doi: <http://dx.doi.org/10.1016/j.clnu.2018.11.001>

Huang, L., Xu, S., Chen, X., Li, Q., Lin, L. C., Zhang, Y., . . . Yang, N. (2020). Delayed Lactogenesis Is Associated with Suboptimal Breastfeeding Practices: A Prospective Cohort Study. *Journal of Nutrition, 150*(4), 894-900. doi: <http://dx.doi.org/10.1093/jn/nxz311>

Kung, M.-S., & Bajorek, B. (2008). Medications in pregnancy: Impact on time to lactogenesis after parturition. *Journal of Pharmacy Practice and Research, 38*(3), 205-208.

Mallan, K. M., Daniels, L. A., Byrne, R., & de Jersey, S. J. (2018). Comparing barriers to breastfeeding success in the first month for non-overweight and overweight women. *BMC pregnancy and childbirth, 18*(1), 461. doi: <https://dx.doi.org/10.1186/s12884-018-2094-5>

Matias, S. L., Nommsen-Rivers, L. A., Creed-Kanashiro, H., & Dewey, K. G. (2010). Risk factors for early lactation problems among Peruvian primiparous mothers. *Matern Child Nutr, 6*(2), 120-133. doi: 10.1111/j.1740-8709.2009.00195.x

Mohebati, L. M., Hilpert, P., Bath, S., Rayman, M. P., Raats, M. M., Martinez, H., & Caulfield, L. E. (2021). Perceived insufficient milk among primiparous, fully breastfeeding women: Is infant crying important? *Matern Child Nutr, 17*(3), e13133. doi: 10.1111/mcn.13133

Nommsen-Rivers, L. A., Chantry, C. J., Peerson, J. M., Cohen, R. J., & Dewey, K. G. (2010). Delayed onset of lactogenesis among first-time mothers is related to maternal obesity and factors associated with ineffective breastfeeding. *Am J Clin Nutr, 92*(3), 574-584. doi: 10.3945/ajcn.2010.29192

Nommsen-Rivers, L. A., Mastergeorge, A. M., Hansen, R. L., Cullum, A. S., & Dewey, K. G. (2009). Doula care, early breastfeeding outcomes, and breastfeeding status at 6 weeks postpartum among low-income primiparae. *Journal of obstetric, gynecologic, and neonatal nursing : JOGNN, 38*(2), 157-173. doi: <https://dx.doi.org/10.1111/j.1552-6909.2009.01005.x>

Preusting, I., Brumley, J., Odibo, L., Spatz, D. L., & Louis, J. M. (2017). Obesity as a Predictor of Delayed Lactogenesis II. *Journal of human lactation : official journal of International Lactation Consultant Association, 33*(4), 684-691. doi: <https://dx.doi.org/10.1177/0890334417727716>

Rocha, B. d. O., Machado, M. P., Bastos, L. L., Barbosa Silva, L., Santos, A. P., Santos, L. C., & Ferrarez Bouzada, M. C. (2020). Risk Factors for Delayed Onset of Lactogenesis II Among Primiparous Mothers from a Brazilian Baby-Friendly Hospital. *Journal of human lactation : official journal of International Lactation Consultant Association, 36*(1), 146-156. doi: <https://dx.doi.org/10.1177/0890334419835174>

Scott, J. A., Binns, C. W., & Oddy, W. H. (2007). Predictors of delayed onset of lactation. *Matern Child Nutr, 3*(3), 186-193. doi: 10.1111/j.1740-8709.2007.00096.x

Sellen, D. W., Thompson, A. L., Hruschka, D. J., Stein, A. D., & Martorell, R. (2004). Early determinants of non-exclusive breastfeeding among Guatemalan infants. *Adv Exp Med Biol, 554*, 299-301. doi: 10.1007/978-1-4757-4242-8_26

Turok, D. K., Sanders, J. N., Eggebroten, J. L., Bullock, H., Gawron, L. M., Yonke, N., . . . Espey, E. (2017). Immediate postpartum levonorgestrel intrauterine device insertion and breast-feeding outcomes: a noninferiority randomized controlled trial. *American Journal of Obstetrics and Gynecology, 217*(6), 665. doi: <http://dx.doi.org/10.1016/j.ajog.2017.08.003>
